# Democratic governance through DAO-based deliberation and voting for inclusive decision making in AI models

**DOI:** 10.1038/s41598-026-40180-8

**Published:** 2026-03-03

**Authors:** Tanusree Sharma, Yujin Potter, Jongwon Park, Yiren Liu, Yun Huang, Sunny Liu, Dawn Song, Jeff Hancock, Yang Wang

**Affiliations:** 1 College of Information Science and Technology, Pennsylvania State University, University Park, 16802 USA; 2 School of Information Sciences, University of Illinois at Urbana-Champaign, Champaign, 61820 USA; 3 Department of Computer Science, University of California, Berkeley, 94720 USA; 4 College of Communication, Stanford University, Stanford, 94305 USA

**Keywords:** Democratic, AI, Decentralized Autonomous Organizations (DAOs), Voting schemes, Deliberation, Computer science, Software

## Abstract

A major criticism of AI development is the lack of transparency, such as, inadequate documentation and traceability in its design and decision-making processes, leading to adverse outcomes including discrimination, lack of inclusivity and representation, and breaches of legal regulations. Underserved populations, in particular, are disproportionately affected by these design decisions. Furthermore, traditional social science techniques such as interviews, focus groups, and surveys struggle to adequately capture user needs and expectations in the digital era, due to their inherent limitations in deliberation, consensus-building, and providing consistent insights. We developed a democratic decision framework utilizing Decentralized Autonomous Organization (DAO) to enable underserved groups to deliberate and reach a consensus on key AI issues. To assess our proposed democratic decision mechanism, we conducted a case study on updating AI model specification based on diverse stakeholders input. We focus on reducing stereotypical biases in text-to-image systems, particularly gender bias in image generation from text prompts. We designed and experimented various governance configurations, including decision aggregation schemes and decision power, to examine how democratic processes could guide updates to AI model. Through a 2 × 2 experimental design, we tested various aggregation schemes (ranked vs. quadratic) and decision power distribution (equal vs. 20/80 differential) in a randomized online experiment (n=177) with participants from the global south and people with disabilities, to study how the varying governance mechanisms impact people’s perceptions of the decision-making processes and resulting output of the AI Model specification. Our results indicate that despite their diverse backgrounds, participants showed convergence in deliberations on several aspects, including user control over image generation, multiple output options for user selection, and the social appropriateness and accuracy of generated images. Our study underscores the importance of use of appropriate governance in democratic decision-making in AI alignment. Notably, the combination of quadratic preference aggregation method which gives minorities more voice and equal decision power distribution, was perceived as a fairer and democratic approach.

**Supplementary Information:**

The online version contains supplementary material available at 10.1038/s41598-026-40180-8.

## Introduction

As AI becomes increasingly prominent, organizations increasingly disconnect from end users. A critique of prior AI development is its limited documentation and traceability about its process and how stakeholders are involved in the in model design, specification, and deployment^1,2^, which in turn may have contributed to negative consequences^3–6^, such as, intensifying discrimination, violating the value of inclusiveness and representation, and breaching legal rules (e.g., privacy, intellectual property licenses, consumer rights), including data that might be obtained without proper consent (e.g., scraped from the Internet^7^). In particular, AI can disproportionately harm underrepresented groups *“along the intersecting axes of race, ethnicity, gender, ability, and position in global hierarchies.”*^8^ Notably, people with disabilities often early adopters of AI - may face heightened risks of potential downstream harms^9–11^. More generally, past work has categorized harms into two types, allocative harms (i.e., opportunities or resources are withheld from certain groups) and representational harms (i.e., certain groups are stigmatized or stereotyped)^3^.

Recent approaches to mitigate these harms center on aligning of large language models (LLMs) to reflect human values^12^. Reinforcement Learning from Human Feedback (RLHF) is one such technique^13^, aiming to fine-tune model outputs using reward-based signals rooted in human preferences^14,15^. However, LLMs still exhibit persistent social biases and toxicity, often inherited from skewed training data distributions^16–18^.

Yet, addressing these systemic issues requires more than technical fixes. It requires active engagement with people, particularly those from underserved communities. Human-centered approaches have traditionally employed interviews, surveys, and focus groups to contextualize perspectives in informing the goals, constraints, and behavior of AI systems ^11,19^. However, traditional social science approaches often fall short in capturing user expectations due to their limitations in continuity in deliberation, achieving consensus in the fast-paced evolution of AI technologies.

**Fig. 1:**
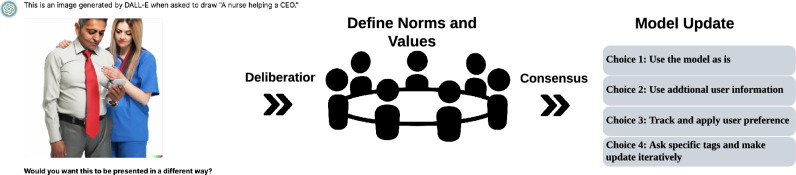
Conceptualization of Democratic Governance Model: A high-level overview of the process, beginning with public deliberation on value-sensitive topics in AI to identify potential improvements for text-to-image models (Case Study). This is followed by a democratic preference aggregation phase, where preference options are drawn directly from the outcomes of the public deliberation. (Note: The image of the nurse and CEO was generated using OpenAI’s DALL.E based on the authors’ prompt (“A nurse helping a CEO”),

Emerging governance models such as polycentric governance and hybrid regulations provide innovative directions that could shape future governance structures^20,21^. Decentralized Autonomous Organizations (DAOs)^22^ are a prime example with an empirical testbed that supports varied structural concepts from management science and community coordination. DAOs are blockchain-based organizations governed by smart contracts and decentralized decision-making, enabling collective governance without centralized control^22^. By leveraging transparent, automated processes with smart contract governance, DAO provides a potential mechanism for accommodating social choice experiments in mainstream AI governance through a computational lens^21,23–26^. However, a fundamental tension exists between participatory decision-making in AI and its global, distributed nature^27^. DAOs offer novel ways to tackle this challenge by incorporating mechanisms such as social choice frameworks, quadratic voting, and liquid democracy^24–26^, while supporting anonymous participation for diverse voices. These approaches further hold promise for solving classical political coordination dilemmas, including preference aggregation and credible commitment issues, which are pivotal for improving representation and accountability in any form of governance (Fig. 1)^28^.

At present, most governance decisions are made unilaterally by technology companies. They often prioritize expert-driven or majority-based decision processes, which may inadvertently marginalize minority viewpoints or underrepresented communities. For instance, OpenAI and other companies often establish internal content policies that determine what models are permitted to say or depict, and subsequently, these policies and requirements are embedded into the training process. While this approach provides centrality and control, it lacks mechanisms for including the preferences and values of affected groups. We argue that aligning model specifications with collective preferences is necessary for socially responsive AI systems. The key challenge remains- **how to establish governance processes that meaningfully involve diverse public in shaping AI behavior.** This work challenges the existing model by proposing computational democratic governance mechanisms with deliberative models to aggregate public preferences for AI.

Our focus is on **how decisions about AI model behavior can be restructured to incorporate broader public input**. We examine these challenges through the **well-known issue as case study - gender bias in text-to-image models**, where models may produce stereotypical associations between professions and gender, for example, depicting a nurse as a woman and a CEO as a man in response to prompts like *“A nurse helping a CEO”.* This case illustrates how generative models can reinforce societal stereotypes and highlights the need for governance mechanisms that allow diverse communities to articulate their expectations for AI outputs in decision making stage.

To address this, we introduce a governance model grounded in the principles of deliberative democracy^29^ which models decision-making through structured discussion and decision power among diverse group participating in AI related decision making. We designed a computational governance design for collective decision-making that draws on theories of democratic and deliberative governance. To facilitate experiment and assess whether this model can function among people, we design computational governance mechanisms with decentralized autonomous organization to run the case study of such governance. Specifically, we draw on decision-making methods from policymaking, electoral systems, and DAO governance to implement four distinct voting schemes.^22,30^. These include preferential voting with weighted aggregation and quadratic voting^31^. While preferential voting with weighted democratic aggregation may inadvertently marginalize minority viewpoints, the quadratic voting system offers a means for minority groups to significantly impact decisions on AI issues that deeply concern or affect them.

We ran a series of randomized control experiments, involving people with disabilities and individuals from the global South, through a $2*2$ experiment design where we constructed the preference aggregation method(ranked voting vs. quadratic) and decision or participation power distribution (equal distribution vs. differential 20/80 distribution). We investigated the impact of different governance configurations (e.g., preference aggregation method, participation power) on the inputs of marginalized groups on their expected design for *“DALLE Text to Image Stereotypical Bias”* Through an online experiment of 177 US internet users, our work underscores the importance of selecting appropriate governance in democratic decision-making in AI alignment. In particular, preference aggregation method - quadratic, combined with equal participation power distribution was perceived as a more equitable and democratic approach, as it amplifies minority voices without privileging dominant groups. By empirically evaluating deliberative AI governance mechanisms, our work contributes to emerging discussions on democratic AI alignment in three key ways.

- First, it provides a systematic experimental assessment of democratic decision framework with decentralized governance for AI decision-making.
- Second, it examines how alternative preference aggregation structures influence stakeholder perceptions of fairness and representation.
- Third, it offers practical design framework for developing governance platforms capable of integrating diverse societal perspectives into AI development processes.

At the same time, our findings highlight potential trade-offs in deliberative governance design. While quadratic preference aggregation may improve minority voices in this case study, it may also risk overrepresenting smaller groups relative to their population proportion. Future research should therefore examine proportional fairness trade-offs and context-specific governance optimization. This work establishes empirical foundation for designing adaptive AI governance mechanisms in which preference aggregation methods and participation power distributions are important governance metrics. Rather than adopting a fixed governance structure, our findings suggest that effective AI governance may require context-sensitive configurations tailored to the goals, expertise, and diversity of participating communities.

### Theoritical grounding for democratic governance of AI

AI governance traditionally focused on translating values and principles into concrete policies, while values define what agents (people or AI) ought to do^32^.

Historically, much of the focus in AI governance research has been at the national and subnational levels^33–35^. More recently, advocacy from civil society, academia, and related stakeholders has increased attention to participatory AI governance to make AI design process more inclusive and equitable^27^. Despite this shift, empirical research on involving stakeholders directly in improving AI performance remains limited with some work, such as, WeBuildAI^36^, which enables collaborative design of community-oriented algorithmic systems, including services like on-demand food donation transport. Similarly, tools like ConsiderIt^37^, which enables structured public deliberation for content moderation decisions, highlight the importance of incorporating user perspectives into decision-making. Research on crowdsourcing further demonstrates that participatory methods can strengthen democratic processes by aggregating individual preferences into collective outcomes while encouraging creativity^38^.

At both theoretical and practical levels, tensions persist between participatory AI governance ideals and the globally distributed nature of AI development^27^. Some works^39^ advocate decentralized governance mechanisms such as governance coordinating committees, international standards, or reliance on existing global legal frameworks^40–42^. Meanwhile, many AI training and refinement processes, such as reinforcement learning depend heavily on labor from developing countries due to pariticipation challenges^27,43^. In designing AI models, the involvement of diverse stakeholders, affected communities, and industry actors in deliberating sensitive issues and shaping model behavior is essential. Another open question concerns the appropriate role in AI itself should play in group decision-making processes^44^.

The rise of decentralized autonomous organizations (DAOs) introduces potential approaches to longstanding coordination problems, dilemmas such as preference aggregation, credible commitments, audience costs, information asymmetry, representation, ^28,45^. The relevance of these theories to the design of digitally-native governance institutions is a critical question^46–48^. The separation of powers in DAOs helps prevent power concentration, enhance transparency, and mitigate organizational gridlock^49^. This is increasingly relevant for AI, where inclusive decision-making is crucial throughout development lifecycle. In this work, we examine DAO-based frameworks for democratic AI governance, focusing especially on preference aggregation and the distribution of decision-making or participation authority.

## Results of case study: gender bias in text-to-image models

### Participant demographics

**Fig. 2:**
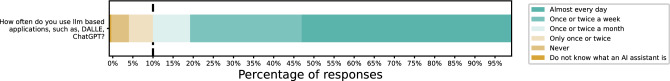
Frequency of study participants’ usage of LLM-based applications. This shows that most participants often use LLMs. $52.2\%$ of respondents reported daily use, followed by $27.7\%$ with weekly use, $9.2\%$ with monthly use, $6.0\%$ who used them only once or twice, $4.3\%$ who reported never using them, and $0.6\%$ who do not know what an AI assistant is.

We had a total of 177 participants in the experiments. Study participants predominantly fell within the 18-24 and 25-34 age brackets, comprising 32.07% and 45.11% of the total, respectively, followed by those aged 35-44 at 11.41%, and 8.70% in the 45-54 age range, etc. In terms of gender distribution, males represented 60.33% while females accounted for 38.59%. Educational backgrounds revealed that 73% held at least a bachelor’s degree, with the remaining participants having attended some college or high school education. The majority identified as Asian/Asian American at 39.67%, closely followed by Black/African American at 29.89%. White/Caucasian participants made up 21.20%, with the remainder being of Hispanic or mixed descent. 50% were from the global south, specifically countries like Bangladesh, India, and Pakistan, while 50% were based in the United States. Regarding technology usage, 78% reported using digital devices very frequently in their daily routines. Another 17% used them frequently, with the remaining participants using them occasionally or not at all. When it came to LLM-based applications, such as DALL.E, ChatGPT 52.2% used these tools almost daily as shown in Fig. 2. 27.7% engaged with them once or twice a week, 9% monthly, and 6% had only used them once or twice. A small fraction, about 5%, had never used such technologies.

Given that our democratic system design for decision-making in AI is deeply rooted in political and citizen sciences, understanding participants’ political ideologies is crucial. 44.57% of our participants identified with the Democratic party, 11.96% associated with the Republican party, 17.39% claimed to be independent or unaffiliated, 2.17% aligned with the Libertarian party, and 7.07% fell into other categories. 15.76% chose not to disclose their affiliation, where the majority of them were from the global south where revealing political ties can be risky due to potential repercussions. When asked about their satisfaction with their country’s political climate, participants’ average Likert scale response was 2.418, indicating a trend towards disagreement and neutrality.

**Fig. 3:**
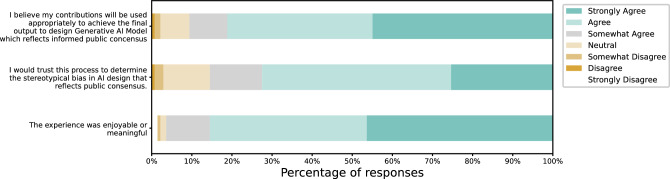
Users’ overall satisfaction with the process. This shows that most of our participants were satisfied with our governance platform. For the first statement “The experience was enjoyable or meaningful,” $46.4\%$ of respondents selected strongly agree, followed by $39.1\%$ with agree, $10.9\%$ with somewhat agree, $1.4\%$ with neutral, $0.7\%$ with somewhat disagree, and $1.5\%$ with strongly disagree. For the second statement “I would trust this process to determine the stereotypical bias in AI design that reflects public consensus,” $25.4\%$ reported strongly agree, $47.1\%$ agree, $13.0\%$ somewhat agree, $11.6\%$ neutral, $7.2\%$ somewhat disagree, and $0.2\%$ disagree. For the third statement “I believe my contributions will be used appropriately to achieve the final output to design Generative AI Model which reflects informed public consensus,” the responses were $44.9\%$ strongly agree, $36.2\%$ agree, $9.4\%$ somewhat agree, $7.2\%$ neutral, $1.4\%$ somewhat disagree, and $0.7\%$ disagree.

### User satisfaction of overall process

Our experiment primarily has two mandatory phases. In the first phase, participants were introduced to a topic related to AI values, specifically focusing on stereotypical biases generated by DALL.E using an unspecified prompt ***“A nurse helping a CEO.”*** They were then asked to express their values concerning this topic. In the subsequent phase, participants voted on a proposal on the same AI value topic stereotypical biases, titled with ***“Update Current AI Model”*** based on their preferences of the images generated by DALL.E.

The overall satisfaction results are depicted in Fig. 3. With a 7-point Likert scale, we assess participants’ satisfaction with the entire process. Out of 177 participants, the feedback indicated a positive experience. Participants found the process enjoyable, with a mean score of 6.23 and a standard deviation of 1.01. They also expressed confidence that their input would be appropriately utilized to shape the final Generative AI Model in a way that reflects informed public consensus, with a mean score of 6.14 and a standard deviation of 1.03. Regarding trust in the process to accurately identify and address stereotypical bias in AI design in line with public consensus, the mean score was 5.80 with a standard deviation of 1.06, suggesting participants generally agreed with the approach. Open-ended responses from our participants further support these findings -*“It seems involving with discussion and at the same concrete with the voting. I was able to share my opinion with different levels of preferences with the system.”*

### Impact of varying voting mechanisms in governance outcome

We measured the impact of different governance mechanisms in the decision related to AI model updates as well as users’ experience and satisfaction with the governance process. We conducted the experiment with two user groups over two rounds (Table 1). Round 1 included participants from Global South countries such as Bangladesh, India, Pakistan, and Nigeria, etc. Round 2 included participants who were blind users living in the United States. First, we conducted statistical significance tests to further assess if different governance mechanisms (treatment conditions) have an impact on the AI model governance outcome. We have two main factors- voting method and voting power in the treatment conditions.

**Table 1:** Summary stats of the ratio of tokens allocated to each voting choice (Choice 1: Use the current models is, Choice 2: Use additional user information, Choice 3: Track and apply user preferences, and Choice 4: Add specific flags and tags in the requests) by users. The ratio is calculated as the percentage of tokens the user allocated to each voting option. For example, if a user allocated 20, 20, 30, 30 tokens for each voting option, the vector for the user would be (0.2,0.2,0.3,0.3).

		Choice 1		Choice 2		Choice 3		Choice 4	
		mean	Std.	mean	std	mean	std	mean	std.
Round 1	Quadratic - same (n: 26)	0.1627	0.1519	0.1596	0.1352	0.3219	0.2086	0.2580	0.1967
	Quadratic -20/80 (n: 24)	0.0901	0.1415	0.1493	0.1549	0.3358	0.2111	0.2646	0.2473
	Ranked - same (n: 27)	0.1478	0.1695	0.2548	0.1614	0.3200	0.1953	0.2100	0.1679
	Ranked - 20/80 (n:25)	0.1133	0.2102	0.2800	0.2120	0.3005	0.2282	0.2342	0.1689
Round 2	Quadratic - equal (n: 20)	0.121	0.172	0.210	0.140	0.282	0.174	0.332	0.201
	Quadratic -20/80 (n: 19)	0.108	0.143	0.210	0.207	0.321	0.183	0.344	0.201
	Ranked - equal (n: 18)	0.053	0.065	0.177	0.165	0.363	0.238	0.407	0.240
	Ranked - 20/80 (n: 18)	0.107	0.167	0.278	0.164	0.381	0.231	0.223	0.127

*ñVoting method and Governance outcome.* To demonstrate the impact of the voting method (quadratic and ranking) in governance outcome for AI model, we separately ran a one-way multivariate analysis of variance (MANOVA) analysis for four-dimensional vectors (i.e., users’ ratios of tokens allocated for four voting choices for decision making on AI model update based on deliberation: *Choice 1: Use the current model as is; Choice 2: Use additional user information; Choice 3: Track and apply user preferences; Choice 4: Add specific flags or tags in the requests*) to evaluate the significance of the voting method to a token allocation of users to the different voting options, which leads to the outcome of certain options being a winner in a proposal (Table 1 presents the descriptive statistics). Given the dependent variable is a four-dimensional vector, we use MANOVA instead of ANOVA, which is used to analyze the relationship with a one-dimensional value. We had a binary parameter, “quadratic,” that has 1 in the quadratic voting method and 0 in the ranked voting method. As a result, as shown in Table 2, the value of Pillai’s Trace test statistics is 0.1100 (*P*-value=0.0222) for round 1 and 0.0583 (*P*-value=0.3714) for round 2, which indicates that a voting method has a statistically significant association with token allocations made by a user only in round 1. We also ran a multiple linear regression, where an independent variable was “quadratic” (i.e., voting method) and dependent variables were users’ ratios of tokens allocated for four voting choices. Our result shows users were more likely to avoid choosing the second option (i.e., Use additional user information) in a quadratic voting mechanism; the result of the coefficient of “quadratic” for each voting choice for round 1 is $-0.0034,$ $-0.1123,$ 0.0180,  and 0.0396,  and for round 2 is 0.0345,  $-0.0178,$ $-0.0716,$ and 0.0228. In other words, when $r_i$ denotes a ratio of tokens that users allocate to choice *i*,  the following relationships with a quadratic voting method for round 1 are met: $r_1 =-0.00335\cdot \texttt {quadratic}+e_1$; $r_2=-0.1123\cdot \texttt {quadratic}+e_2$; $r_3=0.0180\cdot \texttt {quadratic}+e_3$; $r_4=0.0396\cdot \texttt {quadratic}+e_4$.

*Voting power and governance outcome* Similarly, with one-way MANOVA analysis for a vector (token allocations of a user). We had a binary parameter, “same,” that has 1 in the equal voting power condition and 0 in the 20/80 voting power condition. As shown in Table 2, we did not observe a significant relationship between voting power and voting outcome, according to Pillai’s Trace test (value=0.0259, *P*-value=0.6325 for round 1; value=0.0550, *P*-value=0.4034 for round 2).

**Table 2:** MANOVA without interaction.

	Variable	Value	Num DF	Den DF	F value	Pr>F
Round 1	Pillai’s Trace (*quadratic*)	0.1100	4.000	96.000	2.9677	0.0233
	Pillai’s Trace (*same*)	0.0259	4.000	96.000	0.6373	0.6372
Round 2	Pillai’s Trace (*quadratic*)	0.0584	4.000	69.000	1.0689	0.3786
	Pillai’s Trace (*same*)	0.0551	4.000	69.000	1.0056	0.4107

*Interaction of voting method and voting power in governance outcome*

We also evaluated if there is any significant interaction effect between the two main predictor variables, voting power, and voting method. Therefore, we ran a two-way MANOVA analysis for four-dimensional vectors (users’ ratios of tokens allocated for four voting choices). We conducted MANOVA considering the two main predictors with interaction.

**Fig. 4:**
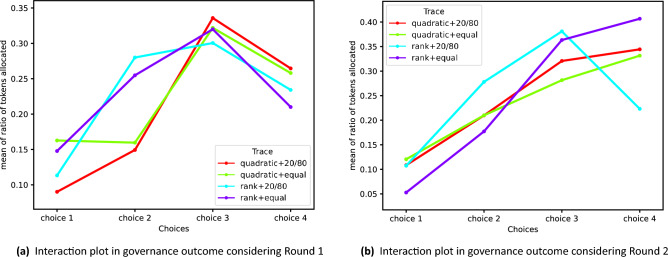
Interaction plot between voting method and voting power distribution. The figures show the average ratio of tokens allocated to each voting option by the voting process design (i.e., voting method and voting power distribution) in rounds 1 and 2. Four choices mean the following: Choice 1: Use the current models, Choice 2: Use additional user information, Choice 3: Track and apply user preferences, and Choice 4: Add specific flags and tags in the requests.

**Table 3:** MANOVA with Interaction.

	Variable	Value	Num DF	Den DF	F value	Pr>F
Round 1	Pillai’s Trace (*quadratic*)	0.0763	4.000	95.000	1.9612	0.1067
	Pillai’s Trace (*same*)	0.0094	4.000	95.000	0.2249	0.9239
	Pillai’s Trace (quadratic*same)	0.0090	4.000	95.000	0.2153	0.9293
Round 2	Pillai’s Trace (*quadratic*)	0.0578	4.000	68.000	1.0432	0.3915
	Pillai’s Trace (*same*)	0.1136	4.000	68.000	2.1790	0.0806
	Pillai’s Trace (quadratic*same)	0.0804	4.000	68.000	1.4869	0.2158

When we run a two-way MANOVA with the interaction between the two variables, voting method, and voting power distribution, as shown in Table 3, we observe that the voting power condition significantly affects the voting outcome in round 2 (Pillai’s Trace value=0.11, *P*-value=0.08). Figures 4 show the average ratio of tokens allocated to each voting option by the voting process design (i.e., voting method and voting power distribution) in rounds 1 and 2. In Fig. 4a, we can see the trend difference between two lines with quadratic and the other two lines with rank. This suggests that whether the voting method was quadratic or ranked significantly affects users’ voting choices. Furthermore, in Fig. 4b, the difference between rank+20/80 and rank+equal is manifested. On the other hand, quadratic+20/80 and quadratic+equal lines exhibit a similar pattern. This implies that a different voting power distribution becomes a pronounced factor in the voting outcome in the ranked voting method condition. These are supported by our statistical analysis, MANOVA.

### Quality of democratic governance decision-making process

**Fig. 5:**
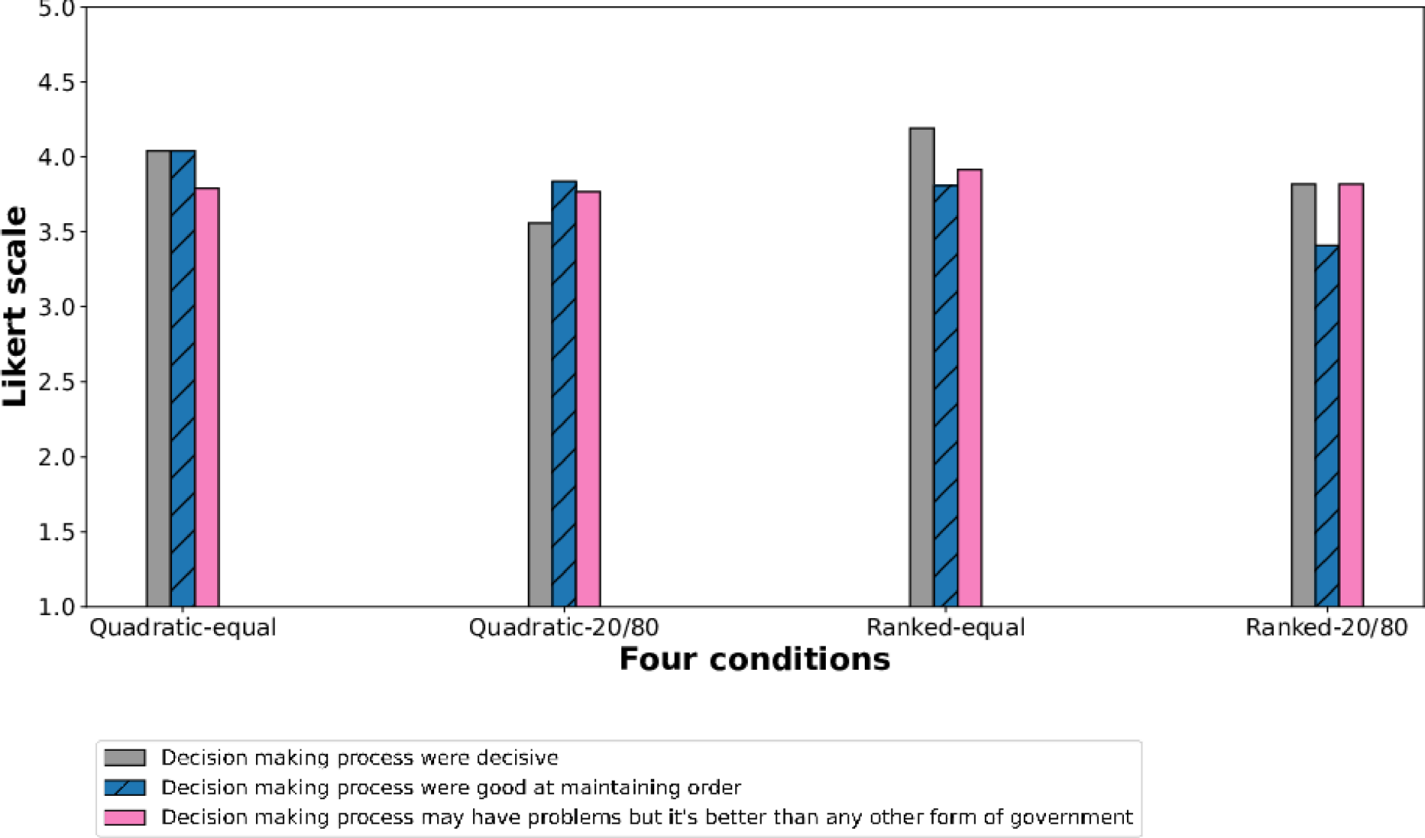
Users’ perception of the decision-making process. This figure shows that participants more highly rated the decision making in the equal voting power condition.

**Fig. 6:**
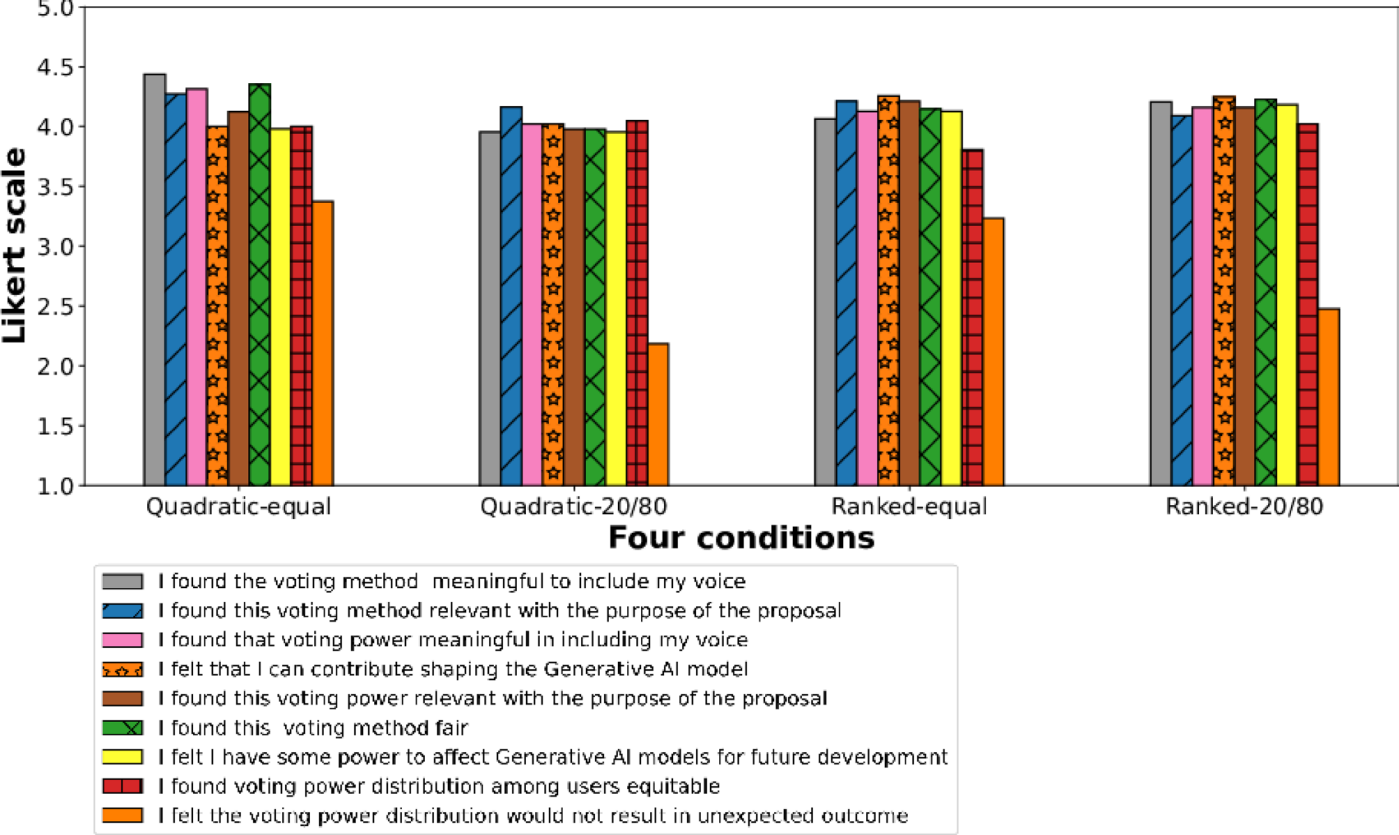
Users’ perception of the quality of voting mechanism in governance decision making. Participants highly agreed that the voting method is meaningful to include their voice, especially under the equal voting power and quadratic voting method conditions. Moreover, participants in the equal voting power condition felt less likely that their voting power distribution could lead to unexpected outcomes than those in the 20-80 voting power condition.

*Users’ General Perception of Voting Mechanism* To gauge users’ attitudes towards the voting process being democratic, we presented several 5-point Likert scale questions (full protocol is included in the supplementary document). We notice participants rated in between agree to strongly agree (mean: 4.14; sd: 0.815) for the Voting method (Weighted ranking/Quadratic) meaningful to include their voice (Fig. 6). A representative quote from a participant further supports this finding, as they expressed- *“I believe that my contributions will be used appropriately to design an AI model that reflects informed public consensus because as a disabled user, I have a unique perspective when it comes to AI and accessibility and design.”* Participants also found that the voting method is relevant to the purpose of the proposal (mean: 4.03, sd:0.92). However, it’s worth noting that some participants expressed uncertainty regarding how AI developers might incorporate these collective decisions– *“ I am skeptical that developers will do the right thing as sometimes they are influenced by other factors such as commercial needs, time constraints or thinking that it is too much work to meet the needs of disabled people or not valuing input from the disabled community as users.”*

More specifically, in equal voting power conditions, participants rated highly for the decision process being decisive (mean=4.12, std=0.74) and were good at maintaining order (mean=3.93, std=1.00) (Fig. 5). As participants quoted *“I found the method useful. Dividing up my votes allowed me to consider the strengths and weaknesses of each presented option–a good metric for measuring the degree of my preference as opposed to just true or false.”* Especially, users who participated in quadratic voting with equal voting power rated highly that the process was good at maintaining order (Fig. 6). Moreover, the users who participated in the equal voting condition tend to believe more that the voting process can be better than any other form of government (mean=3.85, std=0.73). Open-ended responses from participants align with these findings. One participant remarked, *“I had never experienced a quadratic vote before, and I found it incredibly intriguing to assign a weighted value to each item in the selection. It treated everyone equally and no one had special influence. It has been one of the fairest voting and decision-making experiences.”*

**Fig. 7:**
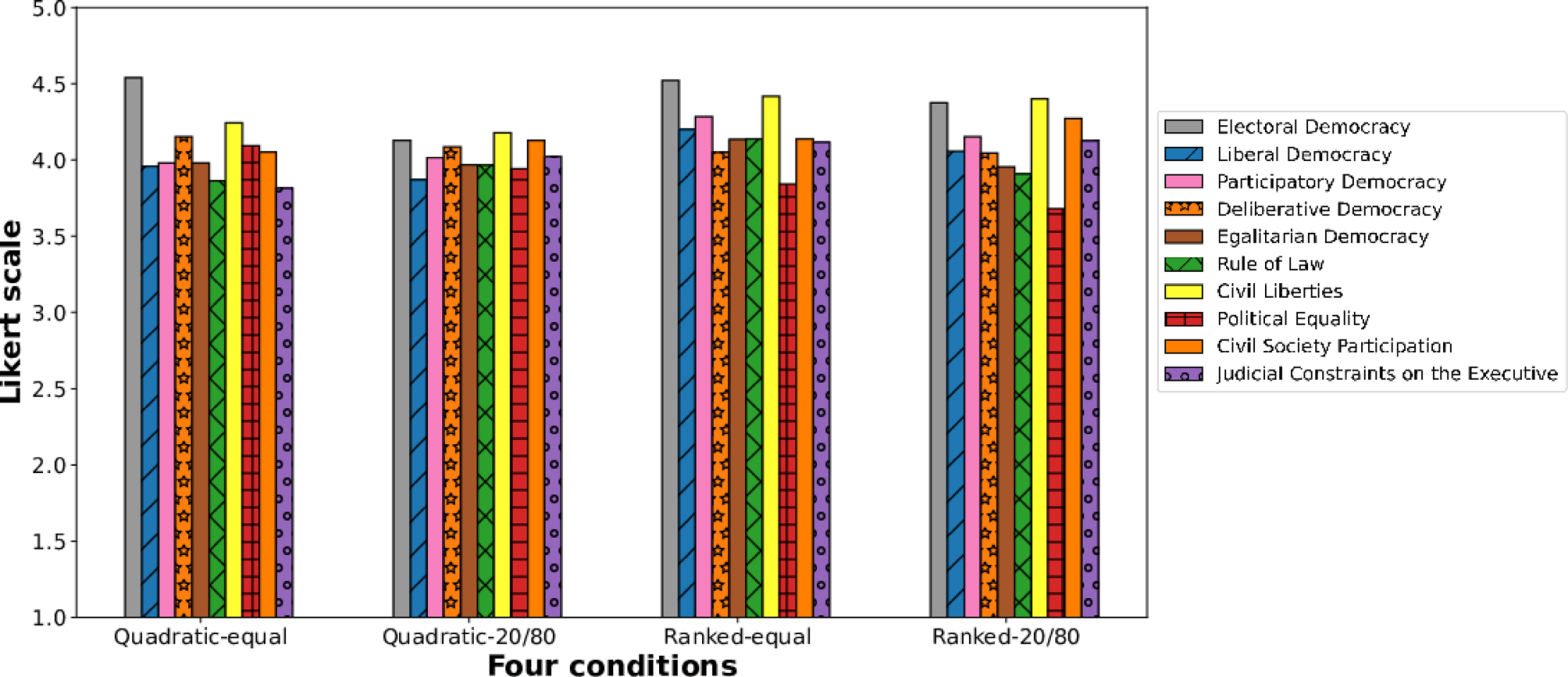
Users’ perception of a voting mechanism (obtained through the V-Dem question lists). Overall, users rated highly for the quality of democracy of the decision-making process in V-Dem measures, which shows our voting process is democratic.

*Quality of decision-making process of different democracies.* Overall users rated highly for the quality of democracy of the decision-making process in a variety of democracy (v-dem) measures, which shows our voting process is democratic (Fig. 7).

To determine whether specific *“voting methods”* were related to users’ perceptions of the process being democratic, we conducted a linear regression analysis. In this analysis, the predictors were the various voting methods, and the dependent variable was users’ attitudes towards the outcome’s quality, as measured by Likert scale questions from the V-Dem measures. Participants who participated in quadratic voting felt fairer than those who participated in ranked voting (linear coefficient$= 0.3931,$ *P*-value$=0.037$). On the other hand, users who participated in the quadratic voting method tended to relatively more perceive that the process was good at maintaining order (linear coefficient= $-0.3297$, P value=0.031).

Moreover, participants rated higher political equality in a quadratic voting mechanism (linear coefficient$= 0.2582,$ *P*-value$=0.058$). The open-ended responses echo this finding, with recurrent themes from participants stating– *“The voting process gives the consumer equal rights to cast their votes and even have an alternative. And I believe this will enable the AI preferences to cover diverse aspects”* On the other hand, users rated lower in liberal democracy, participatory democracy, civil liberties, and judicial constraints on the executive in a quadratic voting mechanism (linear coefficient$=-0.2143,$ *P*-value$= 0.059$; linear coefficient$=-0.2234,$ *P*-value$=0.044$; linear coefficient$=-0.1978,$ *P*-value$=0.022;$ linear coefficient$=-0.2051,$ *P*-value$=0.081,$ respectively). Moreover, participants rated lower in liberal democracy, Participatory Democracy, civil liberties, and Judicial Constraints on the Executive in a quadratic voting mechanism (linear coefficient=$-0.2143$, *P*-value= 0.059; linear coefficient=$-0.2234$, *P*-value=0.044; linear coefficient=$-0.1978$, *P*-value=0.022; linear coefficient= $-0.2051$, *P*-value=0.081, respectively.

Under the same *“voting power”* condition, especially, the quadratic voting method with the same voting power condition, users rated highly for electoral democracy (mean=4.53, std=0.52) for the decision-making process followed by deliberative democracy (mean=4.10, std=0.59), political equality more (mean=3.97, std=0.86). As participants quoted to support the deliberative design and equal consideration in AI governance- *“First as a disabled person, beyond voting, engaging in the public discourse for AI which I use daily for my livelihood and then community discussion features to discuss and share preferences and experiences help me understand the issue more and helped me refine my preferences.”*

We also examined any improvements in participants’ perception of the governance process as democratic and analyzed self-reported responses under equal voting power versus differential voting power conditions. Participants felt that the decision-making process was more decisive under the equal voting power condition (linear coefficient=$-0.4261$, *P*-value=0.000) and more effective in maintaining order (linear coefficient=-0.3056, *P*-value=0.046). They significantly less felt that equal voting power distribution could lead to unexpected outcomes compared to the 20-80 voting power condition (linear coefficient=$-0.9719$, *P*-value=0.000). Additionally, participants in the equal voting power condition reported a stronger sense of electoral democracy (linear coefficient=0.2787, *P*-value=0.001). One participant noted, *“I found the power assigned was an innovative way to participate. Many other processes should do the same. Everyone had the same amount of voice to include, and I didn’t feel there could be some unfair treatment. It was straightforward.”*

*Quality of democracy relating to voting method & voting power* When including the interaction term of voting method and voting power, our results suggest that the two are significantly related to each other in terms of affecting user perception of the quality of the democratic decision-making process. For example, the analysis shows that users tended to feel that a voting method was more meaningful to include their voice when they participated in the quadratic voting mechanism under the equal voting power condition (linear coefficient of quadratic*same= 0.6247, *P*-value=0.003) Alternatively, when users participated in the quadratic voting mechanism with the equal voting power condition, they felt the process was more deliberatively democratic (linear coefficient=0.6029, *P*-value=0.033). In connection to this, a quoted statement emphasizes*“Quadratic Voting on a relative scale allows the minority to receive points which increase the overall transparency.”*

**Fig. 8:**
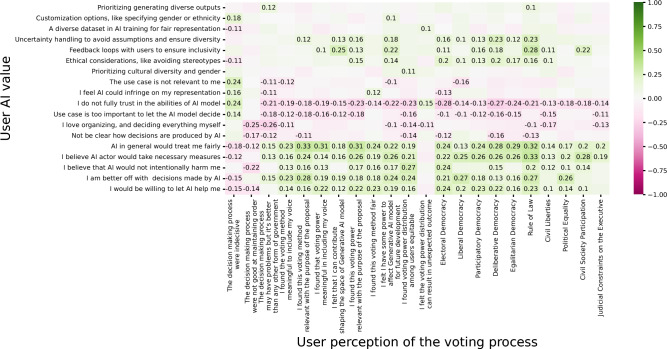
Correlation matrix of users’ perceived quality of democracy (V-Dem Likert Scale) with the predictor’s variables users perceived Value on AI topics (Likert Scale) including construct, such as Trust, perceived fairness, perceived accountability, expected personalization. Green color indicates positive correlation, while pink color indicates negative correlation.

*Relationship Between Users’ Value Towards AI and Their Perceived Value of Democratic System* We investigate the connection between users’ perceptions of AI value topics and their perception of the quality of the process being democratic (Likert subscales from the V-dem measures). Our outcome variables consisted of Likert subscales from the V-dem measures, encompassing Electoral Democracy, Liberal Democracy, Participatory Democracy, Deliberative Democracy, Egalitarian Democracy, Rule of Law, Civil Liberties, Political Equality, Civil Society Participation, and Judicial Constraints on the Executive. If there were more than one item in each subscale. We computed the average scores for each subscale before proceeding with the regression analysis. Our predictor variables were also based on Likert scale questions, capturing users’ perceptions of various AI value constructs, including Trust, Perceived Usefulness, Perceived Fairness, Intention to Adopt, Perceived Accountability, Explainability, and Expected Personalization (complete protocol is in the supplementary document). We began our analysis with a Pearson correlation, followed by the creation of a correlation matrix plot for a more visual representation of the results (Fig. 8).

We have observed that users who exhibit greater trust in AI, particularly with regard to fairness, tend to have a higher perception of the relevance of the voting method and the distribution of voting power (Correlation=0.3291, *p*-value=0.0000; Correlation=0.3108, *p*-value=0.0000). Additionally, they find the distribution of voting power to be more meaningful in terms of including their own voice (Correlation=0.3049, *p*-value=0.0009). Furthermore, these users demonstrate a stronger alignment with the principles of Egalitarian democracy and the Rule of Law (Correlation=0.2896, *p*-value=0.0001; Correlation=0.3138, *p*-value=0.0000). Similarly, individuals who place greater trust in the capabilities of AI models tend to perceive a stronger presence of electoral democracy (Correlation=-0.2808, *p*-value=0.0002). Furthermore, users who prioritize feedback loops with users as crucial for enhancing the diversity and inclusivity of AI images tend to associate more with the concept of the Rule of Law in the voting process (Correlation=0.2867, *p*-value=0.0001). Moreover, users who have a higher level of trust in AI actors, especially those who believe that AI actors would take necessary actions in case of issues with AI decisions or suggestions, tend to perceive a stronger presence of the Rule of Law in the voting process (Correlation=0.3349, *p*-value=0.0000) and greater participation in Civil Society (Correlation=0.2838, *p*-value=0.0002). Lastly, users who feel comfortable with the decisions and suggestions made by AI tend to have a stronger affiliation with the Rule of Law (Correlation=0.2810, *p*-value=0.0002).

### Factors considered by users in balancing diversity and homogeneity for unspecified prompts in text to image generation

To address this research question, we analyzed users’ interactions with both AI and humans. Additionally, we analyzed their Likert scale ratings on AI value constructs, including trust, perceived fairness, expectations, etc.

**Fig. 9:**
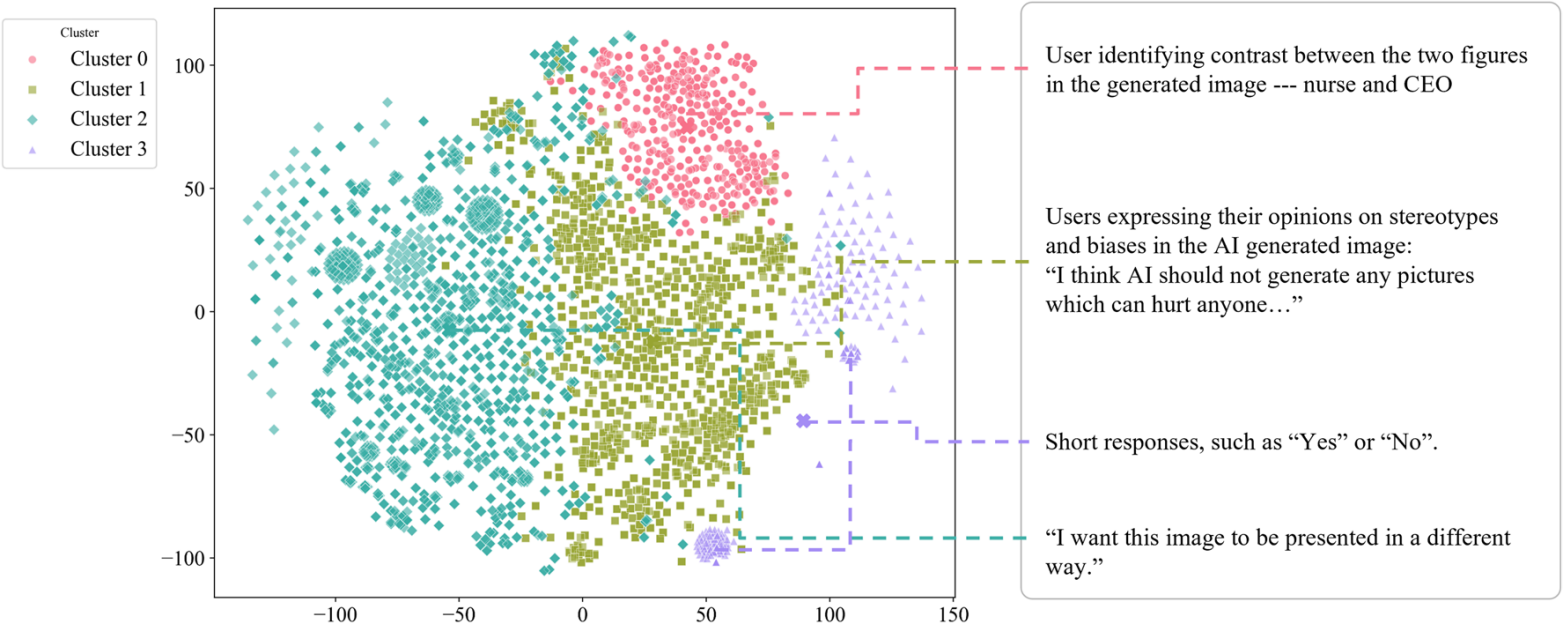
Two-dimensional t-SNE projection of user messages embedded with the OpenAI’s Ada-2 model, reflecting overall themes when conversing with AI. Cluster 0 depicts utterances that explicitly contrast depicted roles or entities, such as in the nurse and CEO example. Cluster 1 consists of users’ reflective comments on social biases and potential harm. Cluster 2 contains users’ instructions to transform or reframe the output. Cluster 3 mostly consists of short responses, including simple acknowledgments and rejections. The dispersion between clusters of the t-SNE plot shows how users often express more scattered and isolated viewpoints when conversing with AI compared to human-human dialogue.

To gain a broader understanding of user interactions with the LLM model in the AI Chat session, we conducted a semantic embedding-based k-means clustering analysis using OpenAI’s Ada-2 embedding model. Employing the Elbow Method, we determined the optimal number of clusters that maximizes the Within-Cluster-Sum-of-Square (WCSS), leading us to choose four clusters (k=4). To visualize and present the clustering results effectively, we generated a two-dimensional plot by applying t-SNE (t-Distributed Stochastic Neighbor Embedding) for dimensionality reduction of the semantic embeddings in Fig. 9. Following the clustering process, We then qualitatively coded the human-AI interaction on a value topic (image content by Generative AI) to better understand the different themes participants discussed during the human-AI chat as well as the discussion with others. We applied thematic analysis to identify the high-level themes and subthemes. Below we presented some themes to show the sneak peak of the outcome.

*Qualitative observation from User Engagement*

We have also found some interesting insights of a qualitative nature that might hold significance for researchers when crafting studies of this kind. In between Human-AI chat and Human-human conversation, there are some subtle differences. For instance, when conversing with AI, users often express more scattered and isolated viewpoints. Conversely, interactions with fellow human users tend to yield more carefully considered responses, frequently featuring conflicting opinions (Fig. 9). To illustrate this, consider a discussion on AI and stereotypes in group conversation:

*“stereotype should not be brought into it to avoid feelings of marginalization on one group over the other. I guess the only way to avoid that is by having some type of standard of things to mention, maybe grammatical standards of sounds, like mentioning objects, adjectives (colors, clothes, face expressions), but I honestly have no definitive answer.”* These findings shed light on the issue of sycophancy observed in Language Model (LM) interactions, where the model often tends to align with and affirm user opinions. This inclination could help explain why dialogues between users and AI typically exhibit fewer instances of conflicting viewpoints compared to interactions between human users^50^.

*Balancing Stereotypes in AI Design.* One theme that stood out was *“Balancing Stereotypes”* Both groups of participants from the United States and the Global south population didn’t oppose stereotypes outright. Instead, they recognized situations where stereotypes might reflect majority scenarios, like more women being in the nursing profession than men which is statistically correct. A representative sample quote from the Global south population - *“Avoiding stereotypes is not always a good thing. Sometimes it may come in handy as it will clearly depict what the user wants and what the user does not want. I see almost 100% of nurses are women. If the user wants a different type of picture then s/he can always ask for it.”* Representative quotes from the United States emphasize more on statistical accuracy given that the current landscape– *“I feel images should convey statistical values, it makes sense that the nurse is a woman as 86% of nurses are women. On the other hand, it would be great if multiple images were displayed to allow the user to choose what fits their use the best. However, the AI should keep in mind the stereotypes and only when prompted to, challenge it.”* However, they emphasized the need for AI to offer multiple outputs for such prompts, ensuring a richer user experience and fostering trust in AI. Few mentioned ethics in this context where they value representation over statistical accuracy.

*“Tolerance Towards the Accuracy of AI-Generated Content.”* Another fact that was different in these two populations is that in the Global South, it implies that they don’t necessarily seek an overwhelming number of diverse generated images that might confuse users. Instead, they appreciate a moderate level of representation and understanding of generated image results that might not be entirely accurate to their depiction all the time. To illustrate this point, one of the representative quotes from Global South– *“ There should be some sort of middle ground. Diverse portrayal of roles to let the people know that there are more than one perspective to an outlook and common representations to not puzzle the user. Having a middle ground for AI.”* Conversely, participants from the United States had different expectations. They wanted the AI to be either entirely accurate or transparent about its inaccuracies. This suggests a lower tolerance among U.S. participants towards AI-generated content. One participant remarked – *“AI decision making be like: 100% or 0% 50% is not a thing for AI unless said so. It lacks the middle ground, unfortunately. I would not prefer middle ground too.”*

*Appropriateness in the social context.*

We also found some unique values and expectations from global South participants. A notable observation was their emphasis on appropriateness within social contexts. To illustrate this point, a quote from Global South, *“It seems unusual for them [image presented nurse helping CEO] to stand so close. If the nurse doesn’t touch the CEO, it would appear more typical. This is a little bit weird if they stand this close. If this CEO was a patient, then it would look normal, I guess, still weird.” * Interestingly, such norms were not present among participants from the United States. In the same line, another prevalent theme was the accuracy of representation in social contexts. Many participants noted that in their countries, the nursing profession is predominantly female, making the AI-generated image a true reflection of their reality. *“AI-generated images should aim to represent a broad spectrum of society. But I don’t know actually. i see in my country most, no all of the nurses are women, so AI is right in my response.”*

*Output multiple images to choose from Balancing Stereotypes in AI Design”* Some other these were predominantly having control to customize the generated images by specifically modifying certain areas of the images according to their preferences. Additionally, there was an expectation for dynamic interactions with the AI, allowing for real-time updates to the generated images. Participants also anticipated the AI to produce multiple image options for a given prompt, granting them the freedom to select their preferred choice. To highlight the sentiment- *“AI-generated images should represent a broad spectrum of society or give options between the spectrum or the common representation. Give a choice so I can pick which image works best for me. I think output multiple images that randomize the sex, race, etc of the people displayed.”* On a more detailed note, some participants wished for a descriptive breakdown of the image and the rationale behind its creation. This would enable them to identify specific areas they’d like to customize further. They likened this process to painting or drawing, where an artist doesn’t finish in one go but revisits, reflects, and adds details over time, mirroring their evolving thoughts and inspirations.

*Users’ concerns of biases in AI image generation.* In addition to varying perceptions, we identified several users who expressed concerns related to the provided value topics, particularly the issue of *stereotypical bias*. Some users worry about the lack of originality in AI-generated images and the potential for AI to create a false sense of perfection or idealized images. Conversely, there were users who worried that excessive tailoring of images by AI to match a person’s profile or preferences could lead to a narrowing of exposure to diverse cultures, races, and experiences. Within the context of inclusivity, users with visual impairments emphasized the importance of AI being inclusive and representative of all individuals, including those with disabilities, as well as people from diverse ethnic and racial backgrounds. In a similar vein, some users raised ethical concerns associated with AI image generation, particularly the potential for manipulation based on pre-existing societal biases. Furthermore, there were concerns that many individuals might lack the necessary understanding to effectively utilize AI technologies, potentially resulting in misuse or underutilization of personalization. Conversely, some users expressed worries about privacy infringement and the misuse of personal data in AI image generation, particularly if excessive personalization were to occur.

## Discussion

### Importing society’s values & deliberative democracy

AI has widespread societal implications on various facets of society, including, healthcare, education, national security, etc^51^. Thus, it is essential that the development and governance of AI technologies involve the public, especially those groups historically marginalized within the technology sector, to ensure responsible development^52,53^. Conventional social science methods, such as interviews, and surveys have constraints and only study public attitudes toward AI technology, often fail to adequately represent collective viewpoints, group deliberation, and regular insights.

In the United States, public opinion has been a significant factor in influencing policy decisions in various areas, including immigration, trade policies, international conflicts, and actions to combat climate change^54^. Given AI’s extensive repercussions, engaging civil society in deliberating about expectations and values becomes crucial for meaningful public influence on AI policy-making. Although the dialogue on the societal impacts of AI and machine learning has started to include technologists and policymakers, public opinion has yet to shape much of these conversations. Therefore, it is crucial not only to gain a deeper understanding of public perceptions regarding AI and its governance but also to devise methods that actively involve the community in the decision-making processes governing AI technologies.

Our study highlights the importance of the decision-making quality in democratic AI governance and emphasizes how deliberative democracy prioritizes the quality of the governance process over the outcome of the governance^55^. Our experimental findings indicate that participants perceived deliberation and voting framework for AI governance both meaningful and effective in including their voices, and relevant in sharing their views in the generative AI model. Notably, those engaged in the governance framework of quadratic voting with equal voting power particularly found the process’s ability to maintain procedural order. Furthermore, the democratic quality of the decision-making process received high ratings across the variety of democracy (v-dem) measures, confirming the democratic integrity of our voting mechanisms.

Preference aggregation method is a standout component in democratic governance framework, which participants found a deciding factor to assess fairness of the democratic process. Specifically, under conditions of participation power of equal, the quadratic preference aggregation method approach was highly rated for promoting electoral democracy, deliberative democracy, and political equality. In particular, our experiment condition with the quadratic preference aggregation method is perceived as equitable, fostering a sense of political equality. This method, when juxtaposed with the principles of deliberative democracy, resonated with participants in AI content of decision-making, making them feel more empowered in decision-making. These result presents a potential of preference aggregation method - quadratic and equal participation power as a potential candidate system to simulate a deliberative democracy for complex value-laden topics, such as stereotypical bias in AI. However, it is crucial to recognize that the appeal of preference aggregation method might differ based on the topic’s nature, especially when issues are controversial or culturally sensitive, warranting further exploration.

### DAO mechanisms as a technical solution for engaging stakeholders in AI

By navigating the complex landscape of both formal and informal political influence, we integrate a constructive methodology with a critical examination of public opinions and attitudes towards generative AI, particularly in relation to the emerging AI application, DALL.E. In the context of the LLM models, open-ended scenarios can be pivotal. The opinions expressed by language models in response to subjective prompts can significantly influence user satisfaction and broader societal implications. In management science, coordination is critical to ensure that resources are used efficiently and that organizations work towards their objectives^56^. In our context, the AI model development considering peoples’ input is crucial where coordination is the key. In addition, digital coordination tools^57,58^, are deemed to be necessary to track progress towards organizational goals. Our developed democratic decision framework, underpinned by the DAO mechanism, offers a promising avenue to actively involve marginalized groups. DAO mechanisms, as digital-first entities, employ mechanisms like initiative proposals, nuanced voting methods, and blockchain-based governance to manage coordination^22^. Participants in our study highly rated our process as being meaningful and that their contribution would be used to achieve the final output to design the AI model. Our results highlight the potential of the voting system to enhance democratic engagement and equality in AI governance, particularly for underserved groups.

However, it is possible that there may be conflicting interests and preferences among underserved groups regarding AI model improvements, particularly concerning stereotypical bias. Our findings reflect some of these differences. For example, generating images of a nurse as a woman was considered statistically accurate by participants from the Global South, aligning with cultural contexts where the majority of nurses are women. In contrast, participants in the United States showed lower tolerance for such representations. Reconciling these conflicting interests and preferences of different underserved groups in AI governance requires a nuanced approach that acknowledges and respects these differences.

This does not mean that all participants need to agree on every topic during deliberation. Deliberation, which involves careful consideration and discussion, has been tested in various countries (e.g., Taiwan) on high-stakes policy questions^59^. In our study, we conducted experiments with mini-publics from diverse backgrounds to reflect the current demographic proportions of blind users and people from the Global South. Our approach conceptualizes decision-making as a form of moral parliament, where participants make decisions under moral uncertainty by imagining representatives of different moral theories who jointly make value decisions^60^.

This study specifically focuses on imitating natural human interaction among participants in a deliberative effort. For instance, during exit interviews, participants emphasized the importance of collective discussion to understand others’ views on socially constructive topics. These exchanges helped them identify feasible compromises or form informed decisions when recording their preferences in voting. Many participants mentioned that despite the discussions, they ultimately made decisions based on their individual preferences. Therefore, regardless of their demographic background, participants commonly exhibited a tendency to uphold their values, learn from others’ perspectives to validate their values or identify similarities and differences, and over time, update their values and preferences.

We also recognize the inherent risk that preference aggregation methods might mirror existing power structures used in decentralized autonomous organizations (DAOs) rather than ideal representations. This study is a first step in this direction, collecting preferences from different demographic groups, training a language model on them, and then aggregating the results within social welfare functions^61^. Future research should further explore the robustness of decisions made by our mini-publics. Specifically, investigating how outcomes of deliberations might vary with changes in the composition of the mini-public. This will enable us to refine this method further to better capture a broad spectrum of perspectives and design a more inclusive approach to AI governance, emphasizing democratic engagement, particularly for underserved groups.

### Future evaluation of democratic framework

Our experiment with one case study - ``Gender Bias in Text-to-Image Models,'' offers early conceptual and empirical insights into how democratic governance can be applied to AI systems. Our experiment involved people from diverse backgrounds, including people with disabilities and the Global South, converged on key LLM design improvements in the text-to-image model, accounting for gender stereotypes. More specifically, our governance tool has been evaluated within a specific scope of OpenAI’s realistic use case through the *“Democratic Input to AI”* initiative, focusing on controversial and high-stakes context, such as, gender and political representation, where LLM output can be controversial^62,63^. As a part of the OpenAI governance initiative, our experiment provides a concrete first step in evaluating stakeholders’ satisfaction with democratic governance mechanisms. Participants involved in the governance decision-making in LLM improvement felt their contributions were meaningful.  This was particularly true in the configurations of quadratic voting with equal power distribution which facilitates minority voices, yielding outcomes perceived as fair and democratic. These results indicate that a DAO-enabled governance process can increase stakeholder satisfaction and equity in AI decision-making compared to centralized approaches, where only a very small group of stakeholders (e.g., frontier AI companies) make the decision. However, we acknowledge that deployment of our system in the wild can further its broader impact.

One practical way to implement this decision framework in LLM applications (e.g., ChatGPT, DALL$\cdot$E, Perplexity, etc) or LLM-powered application (e.g, health chatbot, legal interpretation chatbot) to enable users to challenge and collaboratively deliberate on over AI-generated content. For instance, a*“Challenge this Response”* button could let users flag outputs they considered biased, unclear or harmful. This could open a structured interface where users provide alternative views, engage in a real-time deliberation thread, and vote on LLM improvement suggestions. The system can capture people’s varying perceptions and flags contested outputs for model refinement. Over time, it can aggregate user preferences across domains—politics, health, education, and social issues to inform transparent and participatory AI behavior tuning.

To extend the democratic governance framework beyond the OpenAI pilot, we envision a set of realistic studies modeling contentious AI outputs that may provoke public disagreement. For instance, if an AI system generates misleading vaccine safety information^64,65^,this framework can enable deliberation among patients, public health experts, and ethicists, evaluating outcomes such as perceived legitimacy, diversity of input, and metrics for correction. Another case involves an AI misinterpreting a politician’s video statement, possibly due to its political bias^66,67^, where users may debate contextual cues, tone, and factual framing to test whether participatory governance leads to transparent and trusted content moderation. Such studies will inform partnerships with high-stakes institutions, whereas legal aid firms may use our democratic decision framework to refine AI-generated summaries for pro se litigants ^68^; media outlets can integrate it to audit neutrality in political captioning^69,70^; and civic platforms may apply it to generative summaries of legislation^37,71^. In the future, we aim to evaluate our framework for deliberative refinement model against top-down moderation^72^, non-deliberative crowd voting^29,73^, and expert-only audits^53,74^ to assess trust, transparency, and correction efficacy.

### Scalability and generalization of democratic framework across sectors

The underlying DAO framework is domain-agnostic, meaning that the same governance principles can apply wherever AI impacts stakeholders. For example, recent research in the medical domain shows the potential of DAOs in civic medical data management and decision-making while maintaining transparency. Technologies, such as, smart contract create tamper-proof records to ensure traceability in critical health settings ^75^.

In finance, the platform could be scaled to include diverse stakeholders, such as, customers, compliance officers, and community representatives in governing an AI-driven lending system. A multi-stakeholder DAO could vote on fairness criteria for loan approvals, providing transparency in how decisions are made and reducing the opacity of how AI models are leveraged in banking. By engaging those affected by algorithmic decisions, the system can be tailored to reflect a diversity of values and needs in each sector, from patient privacy to financial inclusion, balancing experts’ views and public participation^76^.

In terms of scalability, we employ a hybrid on-chain/off-chain architecture to handle high volumes of users without sacrificing transparency^77^. We securely record core decisions (votes, proposals) on-chain, while discussion and deliberation can be off-loaded to off-chain channels to ensure responsiveness and low latency even as user counts grow rapidly. This approach benefits from the flexibility of off-chain governance to reduce costs and improve speed, while final outcomes are anchored to the blockchain for verifiability^78^. Moreover, the framework is built to accommodate stakeholder diversity at scale. As evident, decentralized organizations have rapidly grown in number with over 13,000 DAOs operating globally as of 2023, illustrating broad acceptance of such models across domains^79^.

### Ethical and legal considerations in democratic AI governance

Democratic governance through DAO-based deliberation requires careful attention to ethical and legal factors to ensure that the governance process itself upholds inclusivity, fairness while complying with regulations when people’s identity and interaction are involved. Our framework addresses these challenges by minimizing personal data on-chain. Participants used pseudonymized identifiers to engage in deliberation and democratic voting in LLM improvement. Depending on the use case, one can enable a setting to keep sensitive user information (e.g., demographic details or discussion content) off-chain, while decision records remain immutable and transparent. To further ensure privacy, current infrastructure can adapt with emerging solutions like confidential DAOs. This approach encrypt governance data yet maintain transparent, immutable logs^80^. This design help maintain confidence among stakeholders that participation will not expose their personal data and will comply with privacy regulations (e.g., GDPR ^81,82^) and ethical norms.

Another critical consideration is preventing the concentration of power and ensuring fairness within the DAO itself. A known pitfall in blockchain governance systems is the risk of “whale” voters or token-based plutocracy, where a small number of actors accumulate disproportionate influence^22,63,83^. We mitigate this risk through system design, such as, quadratic voting to curb dominance by any single group. For example, quadratic voting gives diminishing returns on additional votes, so that participants must spend votes quadratically to express stronger preferences^24^. This mechanism ensures that minority opinions can still influence outcomes on issues they care deeply about, counterbalancing majority rule. In practice, quadratic voting allowed marginalized voices in our study to impact AI design decisions significantly more than under a traditional one-person-one-vote system. In the future, we will explore alternatives to purely token-based governance, for instance, sortition-based DAOs which enable a rotating panel of randomly chosen participants, similar to *“governance jury,”* for unbiased participant selection, ensuring no clique can continuously dominate decisions^84^. These combined measures are compatible with the current framework to prevent power concentration with fair allocation of voice, advanced voting schemes, and hybrid governance models.

### Limitation

There are several limitations to our approach that merit discussion. Firstly, the design of our group deliberations involved static instructions and some predefined topics if participants were unable to initiate the discussion. To enhance the quality of deliberations to support participants’ understanding of each others’ viewpoints, AI could be integrated to facilitate the deliberation. AI systems can be trained to act as impartial bystanders who aim to bridge differences and help people understand each other’s points of view. Their goal is not to judge participants and they do not take sides on value questions, rather facilitating the deliberation for in-depth conversation.

Secondly, the vulnerability of democratic systems to manipulation by malicious actors poses a significant challenge. These individuals may use misleading statements or harmful rhetoric to skew deliberation outcomes away from the community’s actual values and expectations. One potential mitigation strategy could involve content analysis algorithms to filter out offensive language, though distinguishing contextually appropriate from inappropriate content remains a complex issue, risking the unintended censorship of legitimate discussions. In a similar context, the system’s susceptibility to interference by fake accounts and bots could be addressed in future work through robust identity verification measures, such as biometrics or physical ID checks, to prevent Sybil attacks.

Third, in this study, we set the proposal creation status by AI actors scenario. Future design can allow broader participation in proposal creation, leveraging DAO governance components. This will enable stakeholders—including users, policymakers, and regulators—to generate proposals through direct interactions through communication channels or via dialogue with AI systems. Subsequently, the community can be invited to provide input as “temperature checks,” leading to the formulation of a proposal for voting.

Lastly, the scope of our study was confined to a single application area—generative AI models for text-to-image conversion. In future work, we will assess proposals across a broad spectrum of subjects, encompassing both culturally and politically sensitive topics such as vaccines, immigration, mental health, presidential campaigns, and more. We aim to provide a more comprehensive evaluation of governance quality from users’ perceptions as well as governance outcomes, across various DAO governance mechanisms, with a particular focus on complex and contentious topics.

## Method

Drawing from the HCI literature related to dataset creation^11,85–88^, it’s evident that user engagement in AI innovation is most effective when participants have a clear understanding of the study’s objectives and the tangible results they can expect. By adhering to these principles, our approach to clearly communicate the importance of active participant involvement in AI decision-making at the outset of the study helped effectively guide participants in understanding the AI value topic, fostering discussions, and casting votes. We conducted a between-subjects experiment where participants were exposed to one of four different designs of voting schemes for making governance decisions on AI Text-to-image model topics. The study was approved by the Institutional Review Board (IRB).

Our entire experiment includes three main design components-(1) Human-AI interaction to deliberate on sensitive topics (e.g. Gender Bias in Text-to-Image Models), (2) Group discussion to engage with other to understand collective opinions (3) Governance decisions to guide future LLM model updates.

### Process input & output

**AI Guided Value Topic Discussion** We start by engaging users with an AI Value Topic, related to Stereotypical Bias in Generative AI Models when generating images from text prompts. We started with seed images generated by DALL.E when prompted *A nurse is helping a CEO.* We asked with a simple question *“Would you want this to be presented in a different way? with three options (yes, no, maybe)”* to provoke more thoughts. Through this approach, users were able to disambiguate the intents and values when the AI agent asked them to clarify through natural language conversations on AI value topics and and guides the user to define their norms and expectations. As an input, this stage includes an AI value Topic related to Stereotypical Bias in Generative AI Models when generating text-to-image. AI Prompt design to gauge users’ preferences, and perception of bias.

*Output * This AI-guided value topic discussion provides a set of user open-ended interactions depicting their norms, values, and preferences and self-reported preferences and expectations.

**Group Discussion to Co-Validate Norms** Users then engage in a collective deliberative dialogue process and learn the perspectives of others’ norms in natural language in a Discussion forum in the web application. This way, users can co-validate their values at scale discussing with a mini-public which can allow them to make informed decisions in the democratic process in the next step. We designed the discussion topic based on the pilot experiment of 56 participants from both the USA and the Global South. If participants are unable to introduce a topic on their own, they are encouraged to refer to the suggested topics, such as preference for AI image representation. We designed the discussion topic based on the pilot experiment of 56 participants from both the USA and the Global South.

*Output* This group discussion design provides a set of users’ Interactions depicting their norms, values, and preferences while discussing with others.

**Governance Voting for AI Model Decision** Finally, users participate in the democratic process by voting. We designed experiments to assess varying voting methods and combinations of voting power to examine users’ perception of the quality of the process being democratic in LLM model improvement decisions. We manipulated factors such as voting methods (ranked voting vs. quadratic voting) and voting token distribution (equal distribution vs. 20/80 Pareto distribution). We assessed users’ self-reported quality with the Variety of Democracy (V-Dem) scale^89^ in each condition. The study design was between-subjects, meaning, each user only experienced one treatment condition-design experiment (N = 177, 4 conditions with 44-45 individuals per condition).

*Output* Governance decision provides perceived and actual Quality governance decision outcomes and users’ self-reported quality of the governance outcome in relation to their preferences and values related to the experiment value topic, stereotypical bias in AI image generation in DALL.E.

### Ethical consideration

This study protocol involving human subjects was approved by the Institutional Review Board (IRB) of the Office for the Protection of Research Subjects (OPRS), Office of the Vice Chancellor for Research & Innovation at the University of Illinois at Urbana-Champaign.The IRB protocol approval number is 24401. The institutional and licensing committees involved include the Office of the Vice Chancellor for Research and Innovation and the Office for Protection of Research Subjects at the University of Illinois at Urbana-Champaign. The University of Illinois at Urbana-Champaign Office for the Protection of Research Subjects (OPRS) reviewed the application and determined that the criteria for exemption have been met. This study is not a clinical trial. According to the approved IRB, the Experimental and/or Interventional Research Design includes a Randomized Design and Prospective Social/Behavioral Intervention or Experiment with an AI governance application to coordinate AI users in decision making for large language models design.

During the screening survey, we confirmed that all participants were 18 years or older. The study methods, including surveys, experiments using software to elicit users’ norms of the AI model, user deliberations, and exit interviews, were conducted in accordance with relevant guidelines and regulations. Participants provided informed consent for both the study and the recording of their interactions with the governance process through an online consent form. They were informed of their right to withdraw from the study at any time without any repercussions or loss of benefits.

We informed participants that their quotes would be used in a non-identifiable manner, allowing them to freely express their expectations and values regarding the experimented AI model “Text-to-Image.” This approach, while limiting the extent of quotes or descriptions we could report, facilitated open interactions. We also addressed any questions participants had about the study’s procedure and purpose through the messaging option on their Prolific accounts or via email if they contacted the researchers directly. Transcripts from the exit interviews were pseudonymized and securely stored in a university cloud environment for storage and collaborative coding. Each participant received $40 for their participation, based on an expected study duration of up to 60 minutes.

### Participant recruitment

To help reach a wide range of participants, we recruited through different methods: (1) snowball sampling from our contacts, (2) posting on our Facebook page, and (3) contacting directly by message. We selected participants based on the responses to our screening survey. Respondents were invited to our study if they met all three selection criteria - (1) 18 years or older; (2) use a generative AI tool (e.g., ChatGPT, DALL.E, etc); (c) belonging to underserved groups, including individuals from the global south or those with visual impairments. Participation in our study was voluntary, and participants were allowed to quit anytime. Each participant received a $40 Amazon e-gift card upon completion of the study. The whole interview study lasted about one hour.

### Experimental design

In our experiment, various DAO configurations are independent variables, and the dependent variables are participants’ perceived quality of the process being democratic. We have 2 groups of underserved populations (people with visual impairment, and people from the global south). We followed a between-subject experimental design, where participants from underserved groups were experimented with four different conditions.

*Study type* Democratic Governance system randomly assigned treatments to study subjects. This is also known as an intervention experiment and includes randomized controlled trials. Participants didn’t know the treatment group to which they had been assigned. Study design: $2*2$, 2 factors, 2 levels of 4 combinations; between-subjects design experiment (N = 177, 4 conditions with 43-47 individuals per condition) in each experimental condition.

*Experimental Conditions Design & Rationale* All participants participated in forum discussions in their respective subforums in the treatment groups. Everyone had the option to interact with the AI agent to understand value topics. Everyone had the option to interact with the AI agent to understand the governance mechanism in their respective treatment group. For our particular experience, the proposal options on AI model update (choices 1 - 4 in Fig. 1) were derived from participants’ human-ai and group discussions on stereotypical bias.

*Treatment Condition: Varying Governance Design Framework*

Voting mechanisms and the distribution of voting power play a central role in shaping governance outcomes, as evidenced in both decentralized autonomous organizations (DAOs) and deliberative democratic systems^22,63,90^. To examine how collective preferences might be aggregated for future large language model (LLM) development, we implemented a 2$\times$2 experimental design structured around two variables: voting method and voting power, each with two levels. Although other approaches, such as single choice or approval voting could also be incorporated, doing so would substantially expand the number of treatment conditions and necessitate a much larger participant sample to obtain statistically robust and practically interpretable findings.

For the voting method, weighted ranking and quadratic voting were two levels and for voting power, equal distribution of power and 20/80 (Pareto distribution) were two levels. Thus, there were four treatment conditions- (1) Condition 1: Quadratic Voting Token based (Participants having the same amount of token/voting power); (2) Condition 2: Quadratic Voting 20% population get 80% of the token as early adopters; (3) Condition 3: Ranked voting Token based (participants having the same amount of token/voting power); (4) Condition 4: Ranked voting 20% population get 80% of the token as early adopters. The goal is to evaluate how these different treatment conditions or governance mechanisms, more specifically varying voting methods and combinations of voting power influence users’ perception of the quality of this process being democratic.

*Factor: Voting Method* We chose weighted voting which is widely used in DAO^22^. In this voting method, each user can spread their voting power across any number of choices, from one to all. Their voting power will be divided between their chosen options according to how much weight they attribute to each option by increasing or decreasing the voting power fraction. However, this traditional democratic aggregation can often disfavor strongly held minority views. Therefore we incorporated quadratic voting, which enhances the influence of minorities on issues that are crucial to them. Quadratic voting is a decision-making process where individuals allocate votes based on the intensity of their preferences rather than merely the direction of their preferences. Quadratic voting works by allowing users to “pay” for additional votes on a given matter to express their support for given issues more strongly. For instance, in traditional consensus, if one has 4 voting power, which means 4 votes under the “one token, one vote” principle. However, this system can disadvantage underserved groups who may not have access to many tokens, leading to their voices being overshadowed by those with a larger number of voting power. In contrast, quadratic voting, commonly used in public good funding, calculates the number of votes as the square root of the tokens used—thus, 4 tokens provide 2 votes^91^. This method is considered by DAO practitioners as a way to emphasize the number of voters rather than the size of the voting power. In our experiment, we selected these voting methods to design a governance decision platform for AI stakeholders to deliberate on updates to AI models.

*Factor: Voting Power*The distribution of voting power was another crucial aspect of our governance platform design. Traditional equal distribution might not adequately represent minority groups, whose opinions could be overshadowed by the majority. To address this, we explored the concept of differential power distribution, specifically through the application of the Pareto distribution^92^. This approach is particularly relevant in contexts where AI features are disproportionately beneficial to underserved groups, such as tools for visual impairment. By adopting a combination of voting power, such as equal distribution and 20/80 power distribution, we aimed to evaluate how different democratic aggregations are perceived in terms of fairness by the participants.

## Supplementary Information

Supplementary Information

## Data Availability

Research data are available in this link https://osf.io/q6snh/files/osfstorage?view_only=fb9fd559c9d9472bad1ae7d81dfea3b8

## Code availability

Code Related to the Democratic platform can be found in https://github.com/TanusreeSharma/Inclusive.AI-DAO.
