# Supplementary material for: Democratic governance through DAO-based deliberation and voting for inclusive decision making in AI models
Source: Sci Rep. 2026 Mar 3;16:11792. doi: 10.1038/s41598-026-40180-8 (PMC13066484; doi:10.1038/s41598-026-40180-8)
Supplement: Supplementary file 1 — Supplementary Information [file 41598_2026_40180_MOESM1_ESM.pdf]

Democratic Governance Through DAO-Based Deliberation and Voting for Inclusive Decision Making in AI Model

Tanusree Sharma[1,*], **Yujin Potter**[3], **Jongwon Park**[2], **Yiren Liu** [2], **Yun Huang**[2], **Sunny Liu** [4], **Dawn Song**[3], **Jeff Hancock**[4], **and Yang Wang**[2]

[1]Pennsylvania State University, College of Information Science and Technology, University Park, 16802, USA
[2]University of Illinois at Urbana-Champaign, School of Information Sciences, Champaign, 61820, USA
[3]University of California, Department of Computer Science, Berkeley, 94720, USA
[4]Stanford University, College of Communication, Stanford, 94305, USA
[*]tanusree.sharma@psu.edu

ABSTRACT

Supplementary Document

We utilize Decentralized Autonomous Organizations as a technical intervention to address the challenges around AI governance, more specifically alignment. In this section, we present these three distinct topics, including DAOs, current alignment efforts, and AI Governance concepts.

Decentralized Autonomous Organizations (DAO)

Defnition & History of DAOs

The concept of a DAO has existed since the mid-2010s[1] when DAOs were envisioned as digital alternatives to conventional organizations, promising automation of organizational processes and broader ownership and governance in the digital economy on the basis of a cryptographically secured blockchain[2]. The first DAO, *The DAO*, was originally designed as an investor-driven venture capital fund that relied on voting by investors to disburse funds to proposals submitted by contractors and vetted by curators[3]. It operates as a transparent and democratically structured virtual platform, without physical addresses or formal managerial roles. Despite its potential of launching one of the largest crowdfunded campaigns ever seen[4], it was immediately hacked and drained of $50 million in cryptocurrency[5], highlighting a mismatch between the system's openness and the potential for nefarious actions[4,6,7]. Yet, this should not conflate the broader category of smart contract-based similar technologies, such as Dash governance[8], Digix.io[1], Augur[2], Uniswap[3]. Many of these focused on blockchain-based assets and digital variants of existing socioeconomic instruments such as insurance, exchange markets, and social media[7]. While some researchers argue that DAOs were initially limited to private capital allocation[1,9], there is a growing trend to use DAOs in high-value data, and reputational-based systems[1,10,11]. The deterministic and non-probabilistic nature of smart contracts can be adapted[12] towards these new paradigms based on the refinement of programming logic of organizational rules[13]. Unlike traditional capitalist organizations with undemocratic decision-making processes, where power is concentrated among boards, management, and shareholders, according to Marxist theory[14], DAOs offer a decentralized alternative, allowing for democratic decision-making through consensus protocols[7] and enforces rules for interaction among the members[15].

DAO in Context of Coordination

DAOs enable individuals to coordinate and govern using new technology: smart contracts without centralized control[16]. These give DAOs different competitive advantages in relation to transparency, monitoring and auditing, as well as assurance and expectation. As such, DAOs have different cost functions with respect to a range of key operational and competitive functions within economic coordination, e.g. some have argued that DAOs exist to economize on the costs of trust compared to firms and markets[17]. There is a wealth of academic literature, even if those do not specifically mention the term *"DAO"* by name,

[1]Digix.io is a smart-asset gold-focused coin that seeks to match its value with the price of physical gold.
[2]Augur centers around the prediction markets and betting arenas where financial options and insurance markets can be developed.
[3]Uniswap is a crypto exchange based on smart contracts

including economics theory of the firm[18], public choice theory[19], and voting paradoxes[20]. They analyze the behavior of voters, interest groups, politicians, and bureaucrats in shaping policy and outcomes, which is similar to the structure of DAOs. However, voting paradoxes and the Gibbard-Satterthwaite theorem[21] demonstrate that an individual's voting power can be affected by the voting system's structure and the distribution of voter preferences and that there is no perfect voting system[22] that can consistently and accurately represent the preferences of voters. While prior research has examined the voting power of DAO holders on the Ethereum blockchain[23], however, didn't emphasize participation which is a key element in legitimizing decisions [24]. Our work aims to address this gap by analyzing voting power in relation to participation as well the the level of engagement over time.

Furthermore, coordination in management science is critical to ensure that resources are used efficiently and that organizations work towards the same objectives[25]. Performance metrics and feedback mechanisms[25], as well as computer-based coordination tools[26,27], are deemed to be necessary to track progress towards organizational goals. DAOs as an internet-native organization, and coordination is managed digitally in a fast-paced world. When environmental change is high, organizational systems need to adapt quickly, and this work is typically facilitated by people who focus almost exclusively on coordination as opposed to execution —and that is the role of management, or, put into the language of DAOs, that is the role of community managers and delegates[28]. The specific tasks that require coordination within a DAO and what it means to *"coordinate"* in this context of blockchain require further exploration[29].

DAO in Context of Democracy

DAOs, as digitally constituted organizations, hold significant relevance for political scientists. They provide a novel platform for empirically testing established political science theories. Furthermore, DAOs actively seek expert input to inform their governance design decisions, generating a real-world demand for scholarly exploration in this area. In an era where all facets of society are increasingly digitized, understanding best practices for DAO governance opens doors to reimagining and potentially reengineering current political processes[30]. Political science involves the systematic examination of governance[31,32]. At its core, it revolves around the study of power transfer and allocation in decision-making processes, as well as the emergence and repercussions of diverse governance systems.

Governance questions have puzzled societies and organizations for millennia, with the contested concept of democracy, a system contingent on the will of the people, occupying a central place in this debates[33–35]. In theory, DAOs present innovative opportunities for collective decision-making. However, challenges persist, especially concerning technocracy, which continues to be addressed through adaptable mechanisms like quadratic funding[36] and automated decision-execution protocols. Efforts around avoiding plutocracy or Sybil attacks[37] are ongoing, as common *one-token-one-vote* mechanisms can enable wealthy users to amass a disproportionate number of tokens and, subsequently, an excessive amount of voting power. DAOs are a work in progress as a new governance infrastructure, having issues such as the sensibility of financializing governance and establishing the conditions under which specific voting models are appropriate[38]. Nonetheless, DAOs present an opportunity to tackle the coordination, consensus-building, and power accumulation challenges that exist in centralized organizations, where end users often find themselves marginalized.

DAO as Institute & Firm

The emergence of Decentralized Autonomous Organizations (DAOs) introduces possible solutions for various challenges related to political institutions, including classic coordination dilemmas such as preference aggregation, credible commitments, audience costs, information asymmetry, representation, and accountability[39]. This unique empirical context offers an opportunity for political scientists to examine fundamental theories concerning political institutions and develop innovative theories that can be tested within digital governance. The potential issues associated with tokenized governance prompt intriguing inquiries regarding the design of representative political institutions. Political institutions encompass both formal and informal rules, procedures, and organizations governing individuals' and groups' behavior within a political system[39]. These institutions represent the *rules of the game* or the constraints shaping human interaction[40]. Scholars have engaged in debates about the consequences of different institutional designs, such as the separation of powers, federalism, the strategic configuration of non-democratic institutions, and processes of institutional change. The relevance of these theories to the design of digitally-native governance institutions is a critical question. For instance, the separation of powers in DAOs impacts the prevention of excessive concentration of power, enhance transparency and accountability, or potentially leads to governmental gridlock and indecision[41]. In the context of DAOs, where institutional change can occur rapidly, several factors influence the acceptance of new institutional rules by political elites and the credibility of political actors in upholding these rules[42]. DAOs can safeguard against elite interest capture by implementing mechanisms that prevent non-democratic regimes from using electoral institutions primarily for gathering information about trustworthy ruling elites, thus avoiding performative *window-dressing* to bolster non-democratic regime survival[43]. Recent scholarship has also proposed that blockchain technology, with its disruptive Schumpeterian effects, serves as an institutional technology rather than a general-purpose technology. Furthermore, blockchains themselves can be considered instances of institutional evolution[44].

Summary

DAO encompasses technical components that fundamentally support various structural concepts from fields such as management science, community coordination, political sciences, and more. DAOs hold the potential to address the deficiencies in transparency, consensus-building, coordination, and participation in decision-making by leveraging blockchain governance and smart contracts. We draw upon these theories and the existing DAO literature to define the objectives of my technology deployment.

Governance of Artificial Intelligence

Misalignment & Misrepresentation of AI

There has been a growing interest in exploring the capacity of artificial intelligence, particularly language models (LMs), to emulate human behaviors. One avenue of research investigates whether LMs can replicate outcomes from established human experiments, such as those in cognitive science, social science, and economics[45–49]. Another set of studies explores whether LMs can simulate personas[50–53] similar to our concept of steerability. Through specific case studies, these works assess whether prompting LMs with demographic information (e.g., political identity) leads to human-like responses; for instance, Argyle et al.[51] examine voting patterns and word associations, while Simmons[53] investigates moral biases. In the area of Human and human-LMs alignment, there is a growing body of work aimed at aligning LMs more closely with human values[54–56]. While these efforts acknowledge the subjectivity inherent in the alignment problem, they primarily focus on identifying values to incorporate into models and developing techniques to achieve alignment. There exists inherent variability in what different humans deem as the correct answer. Furthermore, bias, toxicity, and truthfulness have also been extensively studied in the context of NLP systems[49,57–65]. These studies examine properties of LMs, such as bias, toxicity, and truthfulness, with a focus on identifying undesirable outcomes when a well-defined gold standard behavior is in place.

Current AI Govenrnance Effort

As the field of AI continues to evolve and capture the attention of the public and lawmakers[66], the urgency of governance initiatives underscores the growing recognition that AI has the potential to profoundly impact the world, both positively and negatively[67]. Research on AI governance are keeping pace with the ever-changing landscape of policies and technology to address a wide range of AI-related policy challenges[68]. Effective governance can facilitate safety, accountability, and responsible practices in the research, development, and deployment of AI systems. Historically, much of the focus in AI governance research has been at the national and sub-national levels[69–71]. However, research into global AI governance is still in its early stages, though some efforts have been made[72]. Kemp et.al.[73] and some researches advocate for decentralized approaches, such as *"Governance Coordinating Committees,"* global standards, or leveraging existing international legal frameworks[74–76].

There has been an emergence of international collaborations and initiatives aimed at collectively addressing governance challenges. Organizations like the OECD have crafted AI policy frameworks and principles to encourage responsible AI development and foster cooperation among nations (OECD, 2019)[4]. The application of AI in healthcare has introduced unique governance challenges, prompting researchers to dive into issues related to patient data privacy, bias in AI algorithms, and the necessity of robust regulatory frameworks. The *Ethical Principles for AI in Healthcare,* proposed by the American Medical Association (AMA), serves as a guiding document for the ethical development and deployment of AI within the healthcare sector [5]. The European Union's General Data Protection Regulation (GDPR) has played a pivotal role in establishing a precedent for data protection laws that are pertinent to AI applications (Regulation (EU) 2016/679)[6]. Prominent frameworks, such as the principles articulated in the *"Ethics Guidelines for Trustworthy AI"* by the European Commission, underscore the importance of transparency, accountability, fairness, and human agency in the development and deployment of AI [7].

Design Space

AI-based systems are often perceived as *black boxes,* creating significant information imbalances between developers of these systems and consumers and policymakers [8]. AI and algorithmic systems are already playing pivotal roles in shaping decisions across various domains, encompassing both the private and public sectors. For instance, major global platforms like Google and Facebook rely on AI-driven filtering algorithms to control access to information[77]. In the realm of self-driving cars, AI algorithms face the critical challenge of balancing the safety of passengers and pedestrians [9]. Moreover, AI-powered face recognition algorithms hold significant importance in applications such as security and safety decision-making systems.

[4] OECD Principles on Artificial Intelligence. Retrieved from https://www.oecd.org/going-digital/ai/principles/

[5] American Medical Association. https://www.ama-assn.org/practice-management/digital/advancing-health-care-ai-through-ethics-evidence-and-equity

[6] Regulation (EU) 2016/679. (2016). General Data Protection Regulation (GDPR). Retrieved from https://eur-lex.europa.eu/legal-content/EN/

[7] European Commission. Ethics Guidelines for Trustworthy AI. Retrieved from https://ec.europa.eu/digital-single-market/en/news/ethics-guidelines-trustworthy-ai

[8] https://hdsr.mitpress.mit.edu/pub/f9kuryi8/release/8

[9] Source: https://spectrum.ieee.org/self-driving-cars-2662494269

A recent study conducted at Stanford University even highlights an AI algorithm's capacity to discern individuals' sexual orientation on a dating site with remarkable accuracy, sparking concerns among certain segments of society regarding the potential unintended consequences and drawbacks associated with the widespread adoption of these technologies[78].

In order to ensure transparency, accountability, and explainability within the AI ecosystem, it is imperative that governments, civil society, the private sector, and academia come together to discuss governance mechanisms that mitigate risks and potential downsides of AI and autonomous systems while harnessing the full potential of this technology. However, the process of establishing a governance framework for AI, autonomous systems, and algorithms is inherently complex for several reasons. Regulating proactively poses challenges for any industry, particularly given the rapid evolution of AI technologies, which are still in the developmental stages. A global AI governance system must possess the flexibility to accommodate cultural differences and bridge gaps among diverse national legal systems. To address this information gap and facilitate constructive discussions, the literature offers various conceptual frameworks for contemplating AI governance. These frameworks encompass diverse angles and perspectives. Firstly, there is the perspective of *justice and equality,* which scrutinizes the extent to which AI systems can be intentionally designed and operated to embody human values such as fairness, accountability, and transparency. It also seeks to prevent the emergence of new inequalities and biases[79] Another facet is the *Use of Force* angle, which deals with AI-based systems participating in decision-making related to the use of force, especially in cases like autonomous weapons. This perspective raises questions about the necessary level of human control and the allocation of responsibility for AI-generated outputs[80]. The aspect of *Safety and Certification* constitutes a governance mechanism, particularly applicable when AI-based systems have physical manifestations. It focuses on defining and validating safety thresholds[70] Regarding privacy, as AI systems heavily rely on data, there is a need to consider the privacy implications and emerging privacy threats posed by next-generation technologies. This encompasses concerns related to government surveillance and corporate influence over consumers[81]. Finally, the issue of *Displacement of Labor and Taxation* raises questions about the extent to which AI-based machines might replace human jobs or reshape the nature of work. Additionally, it explores the potential impacts of AI on public finances, particularly when robots and AI entities do not contribute to taxation[70].

Why DAO is promising in AI Governance?

Due to issues like lack of inclusiveness, transparency, and integrity, AI-based systems often lack clarity, leading to significant information imbalances among developers and stakeholders, including consumers and policymakers. An effective AI governance system must enhance the collective understanding of AI across various contexts. Decentralized Autonomous Organizations (DAOs), where centralized parties often do not dictate the decision, offer a promising approach to AI governance.

Even when there is a shared understanding of AI technologies and societal consensus, designing effective strategies to address these issues is challenging due to the uncertainty and complexity in the AI ecosystem. Traditional approaches to law and policymaking face limitations in the digital age. Emerging governance models like- polycentric governance[82], hybrid regulation can inspire and guide the development of future governance regimes[83]. DAOs, as seen in applications like CabinDAO [10], exemplify the hybrid institutional governance approach, polycentric governance[82] that can similarly be adopted in AI governance.

System Design

Our system had 4 main components from the user's end to complete the process. (1) Account Creation, (2) Human-AI Interaction, (3) Channel Discussion, and (4) Governance Decision. Here we present a summary of the system implementation.

All components from sign-ins and chatting with AI and others to voting for proposals and filling out surveys are implemented on the website. The website actively communicates with our custom server. We use Web3Auth (third-party) to enable simple signups & sign-ins via email, while also generating a unique MPC wallet for each user. Using Web3Auth's provided features, we Derive the user's blockchain address and enable users to sign authentic vote messages (proves the user has voted). We created two VoteToken in solidity smart contracts. Solidity is an object-oriented programming language designed to run on Ethereum. This is to represent the voting power of users. When users are given voting tokens, smart contracts increase each user's balance (with "mint" function calling) so that they can participate in the voting and consequently we create a proposal. For this voting platform, we utilized Snapshot api which is widely used for offchain governance in DAO to facilitate a transparent voting system. Three key elements are involved in this voting platform: spaces, proposals and votes. Organizations can make spaces. Within these spaces, they can set up admins, moderator, proposal authors and decide on their voting rules and validation strategy. This includes who can vote, who can propose, who can moderate proposal and how many votes are needed for a proposal to win. We created space for each treatment condition, containing a proposal on the value question. As a "validation strategy" we specified anyone can vote with voting power. As an organization, they can set a strategy where anyone can propose including, end users, AI stakeholders, even AI agents.

[10]https://cabin.city/

Sign up/ Sign in We use Web3Auth (third-party) to enable simple signups & sign-ins via email, while also generating a unique MPC wallet for each user. This MPC wallet is later used to sign user's votes for Snapshot proposals, discussed more below. Using Web3Auth's provided features, we can: Derive user's blockchain address and Enable users to sign authentic vote messages (proves the user has voted. First-time user signs up with email SSO via Web3Auth. Web3Auth returns a payload that contains the user's email address and appPubkey, a unique public key assigned by Web3Auth for the user for this app. When the user signs up, the user's email address, appPubkey, and derived blockchain address are sent to the server for storage on database. When user signs up, she is directed to the "Intro" page where she needs to fill out a Typeform-like profile survey to fill in her personal information (we use "Quillforms"). When she completes the form and clicks "Submit," the profile data is sent to the server for storage. The profile setup happens only once after the user signs up. Once the user submits the profile data, the profile setup page is bypassed for subsequent sign-ins.

Human-AI Chat There are two main actions on the AI chat 1. Chat with AI, powered by GPT 2. Take a survey (which is a pre-requisite for voting on value topic) Before the user interacts with the AI, we show "Yes, let's start!" button to initiate the AI chat process. When the user clicks the start button, she is provided with a biased image of "A nurse helping a CEO." with the description "This is an image generated by AI when asked to draw "A nurse helping a CEO."" displayed at the top. The image is followed by a question: "Would you want this to be presented differently?" Below the question, we show three buttons: "Yes," "No," and "Maybe." Based on the user's button click, we display the corresponding chat on the interface and dispatch the chat to the server, which returns an AI response. From there on, whenever the user sends a chat (sent to the server), the AI responds back (from the server). All the chats sent by the user in this AI chat page are aggregated as dialogues for GPT-4 API calls, ie. we feed the user's current chat and the history of the user's chats into GPT-4 for the response. At some point, the user can click on the "Survey" button to complete the survey form consisting of Likert scale questions. We do not set any restriction on when the user can start the survey, as we have not considered how to quantitatively measure and restrict the survey (e.g., wait 10 minutes or 20 chat messages sent). When the user submits the survey data, they is redirected to the discussion page. She can navigate back to the AI chat page at any time.

Human-Human Chat In the discussion , users can chat with other users in their pod. Users can access their pod anytime when using the app, including before filling out the survey on the AI chat page. The discussion page has a chat box that facilitates the live-time chats (through websocket connections described below) and contains a few navigation buttons for convenience.

Consensus Platform Once the user fills out the survey on the AI chat page, they can move to the voting page to read the proposal on her pod's value question and vote. The voting page remains restricted until they completes the survey until the discussion page (is open at all times). At the top of the voting page, the user can read a brief description of what the voting is for, what a proposal is, what impact they have when they votes, what voting mechanism she is on, if she has more votes than others, etc. The actual voting mechanisms are discussed below. When the user allocates votes accordingly and clicks the "Cast Vote" button, this triggers Web3Auth's signing library, which signs a message for Snapshot voting. The voting page also contains a navigation button, a pop-up button to read about the voting mechanism of the user's pod, and a "Need Help with Voting?" button. When the user clicks on the last button, it opens a pop-up that resembles the interface of the AI chat page. On this "Need Help with Voting?" modal, users can ask the AI on voting-related topics. This AI chat connection is appended with different prompts vs. the AI chat page.

Voting Tokens on Optimism We created two VoteToken smart contracts, INCLQ and INCLR, on Optimism that represents the voting power of users. When users are given voting tokens, we call the "mint" function on these smart contracts to increase each user's balance (thus the voting power). After we give all voting tokens to the users, we then create a proposal to reflect the voting power of all users. Why do we create the proposal only after every register? **There is a limitation of Snapshot** When DAOs create proposals, some malicious actors might purchase tokens to sway the votes. For example, a proposal might determine "Who will receive this grant" and a malicious entity can vote to get the grant themselves. Snapshot prevents this malicious behavior by enforcing that all eligible voters are token (vote) holders previous to the proposal creation. Some users are given more tokens than others in the "early" pods, discussed below. We picked Optimism for two main reasons: 1. Cheap gas fee, reliable network with EVM compatibility. 2. Potential integration with Worldcoin's Sign-In feature For (1), cheap gas and EVM compatibility is found in other EVM L2s (rollups) as well. However, with Optimism's stable uptime and reason (2), we picked it as the rollup to deploy these vote token contracts.

Different Themes in Global South and United States

Global South

- Appropriateness in a social context
- AI Representation accuracy in social context

- Explainability of the image content within the image
- Having the control to customize dynamically
- Balancing Stereotypes in AI Design
- Background/Tool fitted for the Generated persona
- Facial expression
- Having a middle ground for AI
- AI Image Representation – Age, ethnicities, Gender
- Training Data Use
- Privacy in Training Data
- User experience for socially different people

United States

- No middle ground accepted for AI Outcome
- Background/Tool fitted for the Generated persona
- Output multiple images to choose from
- Balancing Stereotypes in AI Design
- Having the control to customize dynamically
- Appropriateness
- Training Data Use
- Moderation for bad actors who might deflate AI output

Study Protocol

Human-AI Value Question Measures

Likert Scale Questions after AI Assistant on Value Questions

1. Please indicate your level of agreement (1 to 5 with a scale of Strongly Disagree, Disagree, Neutral, Agree, Strongly Agree) with the following statements related to how AI models should balance possibilities to produce diverse or homogeneous outputs in the context of depicting people:

   a. AI models should prioritize generating diverse outputs to represent a wide range of individuals in various professions.

   b. Customization options, like specifying gender or ethnicity, are vital for inclusive and personalized AI-generated images.

   c. A diverse dataset in AI training is essential to prevent bias and ensure fair representation.

   d. AI developers should prioritize uncertainty handling to avoid assumptions and ensure diverse outputs.

   e. Feedback loops with users are key to enhancing the diversity and inclusivity of AI images.

   f. Ethical considerations, like avoiding stereotypes, are vital for fair AI image depictions of people.

   g. Prioritizing cultural diversity and gender is crucial for AI depictions of individuals in professions.

2. On a scale of 1 to 5 (1: Strongly Disagree; 5: Strongly Agree), please rate your attitude towards the personalization of Generative AI[84]:

a. The use case is not relevant to me.
b. I feel AI could infringe on my representation.
c. I do not fully trust the abilities of the AI model.
d. I am afraid that AI could make a mistake I cannot overrule.
e. The use case is too important to let the AI model decide for me.
f. I love organizing, customizing, deciding, and doing everything myself.
g. I am concerned that it would not be precisely clear how decisions or suggestions are produced by AI.
h. I believe that AI, in general, would treat me fairly when making decisions and suggestions.
i. If I have any problem with the decisions or suggestions of the Generative AI, I believe that the AI actor would take the necessary measures.
j. I believe that AI would not intentionally harm me.
k. I think that I am better off with the decisions and suggestions made by AI.
l. I would be willing to let AI help me.

3. Question Sets for Different Constructs

General

(a) The use case is not relevant to me.
(b) AI could infringe on my representation.
(c) I do not fully trust the abilities of the AI model.
(d) I am afraid that AI could make a mistake I cannot overrule.
(e) The use case is too important for me to let the AI model decide.
(f) I love organizing, customizing, deciding, and doing everything myself.

Trust

(g) AI would not intentionally harm me.

Perceived Usefulness

(h) I think that I am better off with the decisions and suggestions made by AI.

Perceived Fairness

(i) I believe that AI, in general, would treat me fairly when making decisions and suggestions.

Intention to Adopt

(j) I would like to let AI help me.

Perceived Accountability

(k) If I have any problem with the decisions or suggestions of the Generative AI, I believe that the AI actor would take necessary measures.

Explainability

(l) I am concerned that it would not be precisely clear how decisions or suggestions are produced by AI.

Expected Personalization

(m) AI models should prioritize generating diverse outputs to represent a wide range of individuals in various professions.

(n) Customization options, like specifying gender or ethnicity, are vital for inclusive and personalized AI-generated images.

(o) Prioritizing cultural diversity and gender is crucial for AI depictions of individuals in professions.

Quality of Democracy-Survey Measures

Survey measures on Governance components

To understand the participants' perception of the quality of the democratic process, we will use Inglehart and Welzel's (2005)'s Democracy Autocracy Preference (DAP) scale and democratic performance evaluation (DPE) scale. We will also examine the cross-DAO populations' (people with disabilities, global south users, people with color) differences in attitude towards the democratic decision-making process based on the literature of WVS that examine cross-cultural differences in social attitudes and specifically in democratic attitudes, such as Dalton and Ong (2005) used this scale to observe differences in the level of support for democracy in six East Asian countries in comparison to four Western countries.

1. On a scale of 1 to 5 (1= Strongly disagree, 2=Disagree, 3=Neutral, 4=Agree, 5= Strongly Agree), rate your attitude towards the democratic decision-making process that you participated in for the AI context.

   a. The decision-making process was indecisive

   b. The decision-making process was not good at maintaining order

   c. The decision-making process may have problems but it's better than any other form of governance

2. On a scale of 1 to 5 (1= Strongly Agree, 2=Agree, 3=Neutral, 4=Disagree, 5= Strongly Disagree), rate your attitude toward the governance components such as voting method (e.g., quadratic, ranking), voting power, etc?

   a. **Voting Method**

      i. I found the Voting method (Weighted ranking/Quadratic) meaningful to include my voice
      ii. I felt that I could contribute to shaping the space of the AI model
      iii. I found this voting method relevant to the purpose of the proposal

   b. **Participation** (voting power)

      i. I found this voting power /token distribution (e.g., equal power, variable power) meaningful in including my voice
      ii. I felt that I could contribute to shaping the space of AI model
      iii. I found this voting power distribution relevant to the purpose of the proposal

   c. **Fairness**

      i. I found the voting method (Weighted ranking/Quadratic) fair

   d. **Agency**

      i. I felt I had some power to affect change in AI future development

   e. **Equality**

      i. I found voting power distribution among users equitable

   f. **Output**

      i. I felt the voting power distribution could result in an unexpected outcome

V-Dem measures to evaluate democratic decision-making

The Varieties of Democracy (V-Dem) is an approach to conceptualizing and measuring democracy. Below are the survey questions based on the V-Dem methodology for assessing democratic decision-making. Please rate the quality of the decision-making process for the following statement in Likert scales ranging from 1-5, where 1 = "Strongly Disagree", 2 = "Disagree", 3 = "Neutral", 4 = "Agree", and 5 = "Strongly Agree".

1. **Electoral Democracy:**
   - I believe that the voting process was free and fair
   - I felt all users had the right to vote
2. **Liberal Democracy:**
   - I believe AI model will operate independently without interference from the development team
   - I felt free to provide feedback on the AI model update freely without fear of repercussions
3. **Participatory Democracy:**
   - I felt that I had ample opportunities to influence the AI model update process beyond just voting
   - I felt that my feedback matters in the decisions made for AI model updates.
   - I believe AI model update decisions will reflect the needs and preferences of the community.
4. **Deliberative Democracy:**
   - AI model update decisions are made after thorough discussion with the user community
   - I believe developers of this AI model will prioritize user interests over their own preferences
   - There is a culture of open dialogue and discussion in the AI model update community
5. **Egalitarian Democracy:**
   - I felt, regardless of my background, I had equal influence in the AI model update decision process
   - I felt large corporations or specific user groups do not have undue influence over AI model update decision process
   - I believe the development team will take active steps to reduce biases in the AI model updates
6. **Rule of Law:**
   - I believe developers will be held accountable for flaws or biases in the AI model updates after this decision process
   - The decision process treats every user's input equally, regardless of their status
7. **Civil Liberties:**
   - I felt free to express my opinions on the AI model updates without fear
   - I felt free to participate in any community or forum discussing the AI model update decision process
   - Feedback channels for the AI model update the decision process operated without censorship or undue restrictions
8. **Political Equality:**
   - Wealthy individuals do not have more political influence than ordinary citizens
   - All ethnic and religious groups have equal political rights and influence.

9. **Civil Society Participation:**

    - User communities play an active role in shaping AI model update policies
    - The development team actively seeks input from user groups and communities.

10. **Judicial Constraints on the Executive:**

    - The oversight/review body has the power and independence to check the actions of the AI development team
    - AI model update decisions can be challenged and reviewed through established feedback channels

Political Ideology Questions

1. What are the three political issues that matter to you? __________
2. On a scale of 1 (strongly disagree) to 5(strongly agree), please rate your agreement with the following statements, "I am generally satisfied with the current political climate in my country."
3. How important is politics in your daily life?

    a. Very important
    b. Somewhat important
    c. Neutral
    d. Not very important
    e. Not at all important

4. Which political party do you most identify with?

    a. Republic party
    b. Democratic Party
    c. Libertarian party
    d. Green party
    e. Independent / Unaffiliated
    f. Other, please specify, ______
    g. Prefer not to say

5. How would you describe your political orientation?

    a. Very conservative
    b. Somewhat conservative
    c. Moderate
    d. Somewhat liberal
    e. Very Liberal
    f. Not sure
    g. Prefer not to say

Demographics and Filter Questions

General Demographics Questions

1. What is your age range?
    a. 18-24 years
    b. 25-34 years
    c. 35-44 years
    d. 45-54 years
    e. 55-64 years
    f. 65 years and above
2. What is your gender identity?
    a. Male
    b. Female
    c. Non-binary
    d. Prefer not to disclose
    e. Other (please specify)
3. Are you currently enrolled in any educational institution?
    a. Yes
    b. No
4. What is your highest level of education completed?
    a. Less than high school
    b. High school graduate or equivalent
    c. Some college or vocational training
    d. Bachelor's degree
    e. Master's degree
    f. Doctorate or professional degree
    g. Others (please specify)
5. Please select your racial or ethnic background (check all that apply):
    a. White/Caucasian
    b. Black/African American
    c. Asian/Asian American
    d. Hispanic/Latino/Latina
    e. Native American/Indigenous
    f. Pacific Islander

g. Mixed race
h. Other (please specify)

6. What country are you currently residing in? (open-ended text)
7. What is your employment status?
    a. Employed full-time
    b. Employed part-time
    c. Unemployed
    d. Student
    e. Retired
    f. Other (please specify)
8. What is your Household Income?
    a. Under $20,000
    b. $20,000 to $40,000
    c. $40,000 to $60,000
    d. $60,000 to $80,000
    e. $80,000 to $100,000
    f. Over $100,000
9. How frequently do you use technology or digital devices in your daily life?
    a. Very frequently
    b. Frequently
    c. Occasionally
    d. Rarely
    e. Never
10. How often do you use an AI assistant such as ChatGPT?
    a. Almost every day
    b. Once or twice a week
    c. Once or twice a month
    d. Only once or twice
    e. Never
    f. Do not know what an AI assistant is
11. What is your preferred Language of Communication?
    a. English
    b. Other (please specify)
12. How did you hear about this study? (open-ended text)

Screener Questions (Demographics with Visual Impairments and from the Global South)

1. Do you have any visual impairments?
   a. Yes
   b. No
2. Which of the following describes your vision level?
   a. Totally Blind
   b. Some Light Perception
   c. Legally Blind
   d. None of the Above
   e. Other (please specify)
3. Are you from a country located in the Global South? (Countries in Africa, Latin America, Asia, and Oceania)
   a. Yes
   b. No

References

1. Chohan, U. W. The decentralized autonomous organization and governance issues. *Available at SSRN 3082055* (2017).

2. Buterin, V. Daos, dacs, das and more: An incomplete terminology guide. *Ethereum Blog* **6**, 2014 (2014).

3. Mehar, M. I. *et al.* Understanding a revolutionary and flawed grand experiment in blockchain: the dao attack. *J. Cases on Inf. Technol. (JCIT)* **21**, 19–32 (2019).

4. Liu, L., Zhou, S., Huang, H. & Zheng, Z. From technology to society: An overview of blockchain-based DAO. *IEEE Open J. Comput. Soc.* **2**, 204–215 (2021).

5. Dhillon, V. *et al.* The dao hacked. *blockchain enabled applications: Understand blockchain Ecosyst. How to Make it work for you* 67–78 (2017).

6. Morrison, R., Mazey, N. C. & Wingreen, S. C. The dao controversy: the case for a new species of corporate governance? *Front. Blockchain* **3**, 25 (2020).

7. DuPont, Q. Experiments in algorithmic governance: A history and ethnography of "The DAO," a failed decentralized autonomous organization. In *Bitcoin and beyond*, 157–177 (Routledge, 2017).

8. Mosley, L. *et al.* Towards a systematic understanding of blockchain governance in proposal voting: A dash case study. *Blockchain: Res. Appl.* 100085 (2022).

9. Trisetyarso, A., Suparta, W., Kang, C.-H., Abbas, B. S. *et al.* Crypto-governance in stock exchanges: Towards efficient and self-regulated trading system. In *2019 International Conference on contemporary Computing and Informatics (IC3I)*, 192–197 (IEEE, 2019).

10. Myeong, S. & Jung, Y. Administrative reforms in the fourth industrial revolution: the case of blockchain use. *Sustainability* **11**, 3971 (2019).

11. Barbosa, A. C., Oliveira, T. A. & Coelho, V. N. Cryptocurrencies for smart territories: an exploratory study. In *2018 International Joint Conference on Neural Networks (IJCNN)*, 1–8 (IEEE, 2018).

12. Chatterjee, K., Goharshady, A. K. & Pourdamghani, A. Probabilistic smart contracts: Secure randomness on the blockchain. In *2019 IEEE International Conference on Blockchain and Cryptocurrency (ICBC)*, 403–412 (IEEE, 2019).

13. Ciatto, G., Calegari, R., Mariani, S., Denti, E. & Omicini, A. From the blockchain to logic programming and back: Research perspectives. In *WOA*, 69–74 (2018).

14. Bowles, S. & Gintis, H. *Democracy and capitalism: Property, community, and the contradictions of modern social thought* (Routledge, 2012).

15. Hassan, S. & De Filippi, P. Decentralized autonomous organization. *Internet Policy Rev.* **10**, 1–10 (2021).

16. Buterin, V. *et al.* A next-generation smart contract and decentralized application platform. *white paper* **3**, 2–1 (2014).

17. Berg, A. & Berg, C. Exit, voice, and forking. *Berg A Berg C (2020)'Exit, Voice, Forking', Cosmos+ Taxis* **8**, 9 (2017).

18. Williamson, O. E. The theory of the firm as governance structure: from choice to contract. *J. economic perspectives* **16**, 171–195 (2002).

19. Shaw, J. S. Public choice theory. *The concise encyclopedia economics* (2002).

20. Nurmi, H. *Voting paradoxes and how to deal with them* (Springer Science & Business Media, 1999).

21. Benoıt, J.-P. The gibbard–satterthwaite theorem: a simple proof. *Econ. Lett.* **69**, 319–322 (2000).

22. Satterthwaite, M. A. Strategy-proofness and arrow's conditions: Existence and correspondence theorems for voting procedures and social welfare functions. *J. economic theory* **10**, 187–217 (1975).

23. Fritsch, R., Müller, M. & Wattenhofer, R. Analyzing voting power in decentralized governance: Who controls daos? *arXiv preprint arXiv:2204.01176* (2022).

24. Pateman, C. *Participation and democratic theory* (Cambridge University Press, 1970).

25. Faraj, S. & Xiao, Y. Coordination in fast-response organizations. *Manag. science* **52**, 1155–1169 (2006).

26. Fish, R. S., Kraut, R. E. & Leland, M. D. Quilt: A collaborative tool for cooperative writing. In *Proceedings of the ACM SIGOIS and IEEECS TC-OA 1988 conference on Office information systems*, 30–37 (1988).

27. Stokols, D., Misra, S., Moser, R. P., Hall, K. L. & Taylor, B. K. The ecology of team science: understanding contextual influences on transdisciplinary collaboration. *Am. journal preventive medicine* **35**, S96–S115 (2008).

28. Burton, R. M. *et al.* Github: exploring the space between boss-less and hierarchical forms of organizing. *J. Organ. Des.* **6**, 1–19 (2017).

29. Baninemeh, E., Farshidi, S. & Jansen, S. A decision model for decentralized autonomous organization platform selection: Three industry case studies. *Blockchain: Res. Appl.* 100127 (2023).

30. Bernholz, L., Landemore, H. & Reich, R. *Digital technology and democratic theory* (University of Chicago Press, 2021).

31. Goodin, R. E. & Klingemann, H.-D. *A new handbook of political science* (Oxford University Press, 1998).

32. Roskin, M. G. Bridging the european divide: Middle power politics and regional security dilemmas. *Perspectives on Polit.* **3**, 957–957 (2005).

33. Rousseau, J.-J. The social contract (1762). *Londres* (1964).

34. Dahl, R. Democracy and its critics yale university press. *New Haven & Lond.* (1989).

35. Landemore, H. *Democratic reason: Politics, collective intelligence, and the rule of the many* (Princeton University Press, 2012).

36. Buterin, V., Hitzig, Z. & Weyl, E. G. A flexible design for funding public goods. *Manag. Sci.* **65**, 5171–5187 (2019).

37. Douceur, J. R. The sybil attack. In *International workshop on peer-to-peer systems*, 251–260 (Springer, 2002).

38. Sharma, T. *et al.* Unpacking how decentralized autonomous organizations (daos) work in practice. *arXiv preprint arXiv:2304.09822* (2023).

39. Hall, P. A. & Taylor, R. C. Political science and the three new institutionalisms. *Polit. studies* **44**, 936–957 (1996).

40. North, D. C. Institutional change: a framework of analysis. In *Social rules*, 189–201 (Routledge, 2018).

41. De Montesquieu, C. *Montesquieu: The spirit of the laws* (Cambridge University Press, 1989).

42. Weingast, B. R. The economic role of political institutions: Market-preserving federalism and economic development. *The J. Law, Econ. Organ.* **11**, 1–31 (1995).

43. Gandhi, J. *et al.* Political institutions under dictatorship. *Camb. Univ. Press. New York* (2008).

44. Davidson, S., De Filippi, P. & Potts, J. Blockchains and the economic institutions of capitalism. *J. Institutional Econ.* **14**, 639–658 (2018).

45. Uchendu, A., Ma, Z., Le, T., Zhang, R. & Lee, D. Turingbench: A benchmark environment for turing test in the age of neural text generation. *arXiv preprint arXiv:2109.13296* (2021).

46. Karra, S. R., Nguyen, S. T. & Tulabandhula, T. Estimating the personality of white-box language models. *arXiv preprint arXiv:2204.12000* (2022).

47. Aher, G., Arriaga, R. I. & Kalai, A. T. Using large language models to simulate multiple humans. *arXiv preprint arXiv:2208.10264* (2022).

48. Binz, M. & Schulz, E. Using cognitive psychology to understand gpt-3. *Proc. Natl. Acad. Sci.* **120**, e2218523120 (2023).

49. Srivastava, A. *et al.* Beyond the imitation game: Quantifying and extrapolating the capabilities of language models. *arXiv preprint arXiv:2206.04615* (2022).

50. Park, J. S. *et al.* Social simulacra: Creating populated prototypes for social computing systems. In *Proceedings of the 35th Annual ACM Symposium on User Interface Software and Technology*, 1–18 (2022).

51. Argyle, L. P. *et al.* Out of one, many: Using language models to simulate human samples. *Polit. Analysis* **31**, 337–351 (2023).

52. Jiang, Z., Xu, F. F., Araki, J. & Neubig, G. How can we know what language models know? *Transactions Assoc. for Comput. Linguist.* **8**, 423–438 (2020).

53. Simmons, G. Moral mimicry: Large language models produce moral rationalizations tailored to political identity. *arXiv preprint arXiv:2209.12106* (2022).

54. Askell, A. *et al.* A general language assistant as a laboratory for alignment. *arXiv preprint arXiv:2112.00861* (2021).

55. Ouyang, L. *et al.* Training language models to follow instructions with human feedback. *Adv. Neural Inf. Process. Syst.* **35**, 27730–27744 (2022).

56. Glaese, A. *et al.* Improving alignment of dialogue agents via targeted human judgements. *arXiv preprint arXiv:2209.14375* (2022).

57. Nadeem, M., Bethke, A. & Reddy, S. Stereoset: Measuring stereotypical bias in pretrained language models. *arXiv preprint arXiv:2004.09456* (2020).

58. Dhamala, J. *et al.* Bold: Dataset and metrics for measuring biases in open-ended language generation. In *Proceedings of the 2021 ACM conference on fairness, accountability, and transparency*, 862–872 (2021).

59. De-Arteaga, M. *et al.* Bias in bios: A case study of semantic representation bias in a high-stakes setting. In *proceedings of the Conference on Fairness, Accountability, and Transparency*, 120–128 (2019).

60. Brown, T. *et al.* Language models are few-shot learners. *Adv. neural information processing systems* **33**, 1877–1901 (2020).

61. Gao, L. *et al.* A framework for few-shot language model evaluation. *Version v0. 0.1. Sept* (2021).

62. Liang, P. *et al.* Holistic evaluation of language models. *arXiv preprint arXiv:2211.09110* (2022).

63. Xu, J. *et al.* Bot-adversarial dialogue for safe conversational agents. In *Proceedings of the 2021 Conference of the North American Chapter of the Association for Computational Linguistics: Human Language Technologies*, 2950–2968 (2021).

64. Perez, E. *et al.* Red teaming language models with language models. *arXiv preprint arXiv:2202.03286* (2022).

65. Ganguli, D. *et al.* Red teaming language models to reduce harms: Methods, scaling behaviors, and lessons learned. *arXiv preprint arXiv:2209.07858* (2022).

66. Zhang, D. *et al.* The ai index 2021 annual report. *arXiv preprint arXiv:2103.06312* (2021).

67. Dafoe, A. Ai governance: a research agenda. *Gov. AI Program, Futur. Humanit. Institute, Univ. Oxford: Oxford, UK* **1442**, 1443 (2018).

68. Cave, S. & ÓhÉigeartaigh, S. S. Bridging near-and long-term concerns about ai. *Nat. Mach. Intell.* **1**, 5–6 (2019).

69. Calo, R. Artificial intelligence policy: a primer and roadmap. *UCDL Rev.* **51**, 399 (2017).

70. Gasser, U. & Almeida, V. A. A layered model for ai governance. *IEEE Internet Comput.* **21**, 58–62 (2017).

71. Scherer, M. U. Regulating artificial intelligence systems: Risks, challenges, competencies, and strategies. *Harv. JL & Tech.* **29**, 353 (2015).

72. Butcher, J. & Beridze, I. What is the state of artificial intelligence governance globally? *The RUSI J.* **164**, 88–96 (2019).

73. Erdélyi, O. J. & Goldsmith, J. Regulating artificial intelligence: Proposal for a global solution. In *Proceedings of the 2018 AAAI/ACM Conference on AI, Ethics, and Society*, 95–101 (2018).

74. Cihon, P. Standards for ai governance: international standards to enable global coordination in ai research & development. *Futur. Humanit. Institute. Univ. Oxf.* 340–342 (2019).

75. Maas, M. M. Aligning ai regulation to sociotechnical change. *Oxf. Handb. on AI Gov. (Oxford Univ. Press. 2022 forthcoming)* (2021).

76. Wallach, W. & Marchant, G. E. An agile ethical/legal model for the international and national governance of ai and robotics. *Assoc. for Adv. Artif. Intell.* (2018).

77. Akter, S. *et al.* Algorithmic bias in data-driven innovation in the age of ai (2021).

78. Wang, Y. & Kosinski, M. Deep neural networks are more accurate than humans at detecting sexual orientation from facial images. *J. personality social psychology* **114**, 246 (2018).

79. Manyika, J., Silberg, J. & Presten, B. What do we do about the biases in al. *Harv. Bus. Rev. Oct.* **25** (2019).

80. Margulies, P. The other side of autonomous weapons: Using artificial intelligence to enhance ihl compliance. *Lieber Inst. for Law Land Warf. US Mil. Acad. at West Point, The Impact Emerg. Technol. on Law Armed Confl. (Oxford Univ. Press. Eric Talbot Jensen ed., 2018, Forthcoming), Roger Williams Univ. Leg. Stud. Pap.* (2018).

81. Manheim, K. & Kaplan, L. Artificial intelligence: Risks to privacy and democracy. *Yale JL & Tech.* **21**, 106 (2019).

82. Ostrom, E. Beyond markets and states: polycentric governance of complex economic systems. *Am. economic review* **100**, 641–672 (2010).

83. Weber, R. H. Realizing a new global cyberspace framework. *Normative Foundations Guid. Princ.* (2015).

84. Meurisch, C. *et al.* Exploring user expectations of proactive ai systems. *Proc. ACM on Interactive, Mobile, Wearable Ubiquitous Technol.* **4**, 1–22 (2020).
